# Key novelties in the evolution of the aquatic colonial phylum Bryozoa: evidence from soft body morphology

**DOI:** 10.1111/brv.12583

**Published:** 2020-02-07

**Authors:** Thomas F. Schwaha, Andrew N. Ostrovsky, Andreas Wanninger

**Affiliations:** ^1^ Department of Evolutionary Biology, Integrative Zoology, Faculty of Life Sciences University of Vienna Vienna 1090 Austria; ^2^ Department of Palaeontology, Faculty of Earth Sciences, Geography and Astronomy University of Vienna Vienna 1090 Austria; ^3^ Department of Invertebrate Zoology, Faculty of Biology Saint Petersburg State University Saint Petersburg 199034 Russia

**Keywords:** Lophotrochozoa, Trochozoa, Spiralia, soft tissue morphology, novelties, phylogeny, Myolaemata, colonial integration, ground pattern, character evolution

## Abstract

Molecular techniques are currently the leading tools for reconstructing phylogenetic relationships, but our understanding of ancestral, plesiomorphic and apomorphic characters requires the study of the morphology of extant forms for testing these phylogenies and for reconstructing character evolution. This review highlights the potential of soft body morphology for inferring the evolution and phylogeny of the lophotrochozoan phylum Bryozoa. This colonial taxon comprises aquatic coelomate filter‐feeders that dominate many benthic communities, both marine and freshwater. Despite having a similar bauplan, bryozoans are morphologically highly diverse and are represented by three major taxa: Phylactolaemata, Stenolaemata and Gymnolaemata. Recent molecular studies resulted in a comprehensive phylogenetic tree with the Phylactolaemata sister to the remaining two taxa, and Stenolaemata (Cyclostomata) sister to Gymnolaemata. We plotted data of soft tissue morphology onto this phylogeny in order to gain further insights into the origin of morphological novelties and character evolution in the phylum. All three larger clades have morphological apomorphies assignable to the latest molecular phylogeny. Stenolaemata (Cyclostomata) and Gymnolaemata were united as monophyletic Myolaemata because of the apomorphic myoepithelial and triradiate pharynx. One of the main evolutionary changes in bryozoans is a change from a body wall with two well‐developed muscular layers and numerous retractor muscles in Phylactolaemata to a body wall with few specialized muscles and few retractors in the remaining bryozoans. Such a shift probably pre‐dated a body wall calcification that evolved independently at least twice in Bryozoa and resulted in the evolution of various hydrostatic mechanisms for polypide protrusion. In Cyclostomata, body wall calcification was accompanied by a unique detachment of the peritoneum from the epidermis to form the hydrostatic membraneous sac. The digestive tract of the Myolaemata differs from the phylactolaemate condition by a distinct ciliated pylorus not present in phylactolaemates. All bryozoans have a mesodermal funiculus, which is duplicated in Gymnolaemata. A colonial system of integration (CSI) of additional, sometimes branching, funicular cords connecting neighbouring zooids *via* pores with pore‐cell complexes evolved at least twice in Gymnolaemata. The nervous system in all bryozoans is subepithelial and concentrated at the lophophoral base and the tentacles. Tentacular nerves emerge intertentacularly in Phylactolaemata whereas they partially emanate directly from the cerebral ganglion or the circum‐oral nerve ring in myolaemates. Overall, morphological evidence shows that ancestral forms were small, colonial coelomates with a muscular body wall and a U‐shaped gut with ciliary tentacle crown, and were capable of asexual budding. Coloniality resulted in many novelties including the origin of zooidal polymorphism, an apomorphic landmark trait of the Myolaemata.

## I. INTRODUCTION

**Figure 1:**
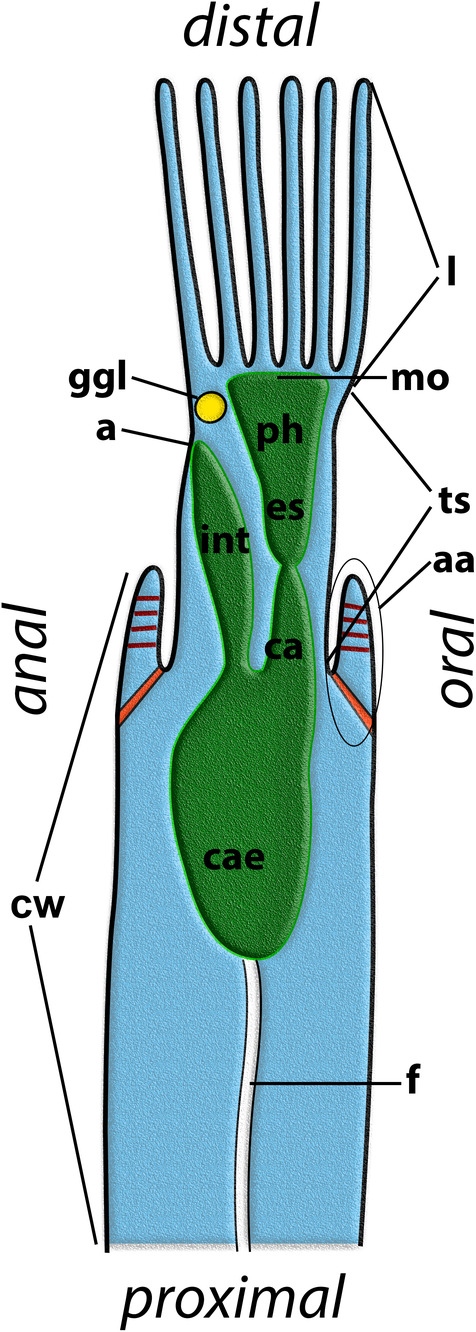
Schematic overview of a generalized bryozoan zooid showing major organ systems like the lophophore and digestive tract, and the general axis orientation for the polypide. Abbreviations: a, anus; aa, apertural area; ca, cardia; cae, caecum; cw, cystid wall; es, oesophagus; f, funiculus; ggl, cerebral ganglion; int, intestine; l, lophophore; mo, mouth opening; ph, pharynx; ts, tentacle sheath.

Bryozoa (Ectoprocta) is an aquatic phylum that comprises more than 6000 described recent and 15000 fossil species of epibiotic, active suspension‐feeding coelomate invertebrates (Gordon, Taylor & Bigey, 2009; Bock & Gordon, 2013). Bryozoans are known from the beginning of the Ordovician and represent major components of most benthic ecosystems from the intertidal to abyssal depths exceeding 8000 m. They are colonial and consist of modules (zooids) that are usually less than 1 mm long. Each zooid comprises a polypide (retractile ciliated tentacular crown associated with a U‐shaped gut and retractor muscles) and a cystid (body wall) (Fig. 1). The tentacle crown is conventionally termed the lophophore and the latter is connected to the cystid *via* an everting part of the body wall, the tentacle sheath (Ryland, 1970, 1976, 2005; Boardman, Cheetham & Cook, 1983; McKinney & Jackson, 1989; Reed, 1991). The latter is sometimes referred to as the ‘introvert’, although this is not totally accurate because the tentacle sheath is not the only introvertable area (see Schwaha, 2019*b*). Due to the U‐shaped gut, an oral and anal side of each zooid can be differentiated. At the level of the polypide, the side facing the substrate is usually referred to as proximal and the opposite as distal (Fig. 1). Within the different bryozoan subclades, the terminology can vary depending on the colony form and zooidal arrangement (Boardman, Cheetham & Cook, 1983; Cheetham & Cook, 1983).

Despite this common ground pattern, bryozoans show very high morphological diversity of both skeletal and soft tissues, calling for a comparative and evolutionary analysis. In fact, the high diversity of bryozoans through geological time is an important indicator of their evolutionary success. Three larger taxa are commonly recognized among Bryozoa: (*i*) the solely freshwater‐inhabiting Phylactolaemata; (*ii*) the marine Stenolaemata with the only surviving taxon Cyclostomata; and (*iii*) the predominantly marine and morphologically most diverse Gymnolaemata (Bock & Gordon, 2013). Allman's (1856) original classification divided Bryozoa into Phylactolaemata [*phylasso* – to guard, *laimos* – throat (referring to the epistome, a ciliated flap ‘guarding’ or covering the mouth opening] and Gymnolaemata [‘naked throats’ (*gymnos* – naked) with respect to the missing epistome] which initially included three groups defined by Busk (1852) as Cyclostomata (*cyclo* – ring, *stoma* – mouth), Ctenostomata (*ktenos* – comb) and Cheilostomata (*cheilos* – lip; sometimes used as Gymnolaemata *sensu lato*, e.g. Jebram, 1986*a*,*b*). Cyclostomata were renamed into Stenolaemata [term coined by Borg (1926) from *stenos* – narrow], subsequently modified to Stenostomata by Marcus (1938) – a distinct clade that in addition to cyclostomes also includes four Palaeozoic groups. Gymnolaemata, therefore, were confined to Cteno‐ and Cheilostomata (Borg, 1926; Silén, 1942). An alternative name for the current Gymnolaemata is Eurystomata (from *euros* – wide; Marcus, 1938) that, with few exceptions, is now abandoned and should not be used to avoid confusion.

Phylactolaemata is a small clade of only about 80–90 described extant species. Their zooids with non‐calcified walls are larger than in marine forms (diameter of the tentacle crown reaches 1 × 1.5 mm). Almost all possess a horseshoe‐shaped lophophore and all have statoblasts, a dormant dispersal stage (Wood, 1983). Other bryozoans have a circular tentacle crown and their zooids are usually much smaller than in phylactolaemates. Stenolaemata (Cyclostomata) is represented by about 850 recent species which possess a calcified skeleton and a number of unique features such as the membranous sac and polyembryony (Nielsen & Pedersen, 1979; Reed, 1991; Mukai, Terakado & Reed, 1997).

Two distinct groups are recognized among the Gymnolaemata: the uncalcified and paraphyletic ‘Ctenostomata’ and the calcified Cheilostomata that comprise most post‐Paleozoic bryozoan species (Cheetham & Cook, 1983). Both Cyclostomata and Cheilostomata are considered rooted among ancient ‘ctenostome‐like’ bryozoans, suggesting at least two independent calcification events in the Paleozoic and Mesozoic (Larwood & Taylor, 1979; Taylor & Larwood, 1990; Ernst & Schäfer, 2006; Taylor & Waeschenbach, 2015).

Traditionally, phylogenetic classifications of Bryozoa were predominantly based on external morphology (mainly skeletal) and have resulted in a number of contradicting scenarios that were used to explain interrelationships of the different clades [for reviews, see Boardman *et al*. (1983), Todd (2000) and Ostrovsky (2013*a*)]. Attempts to use soft tissue characters for this purpose are rare (e.g. Borg, 1926; Soule, 1954; Boardman & McKinney, 1985; Schäfer, 1985; Jebram, 1986*a*,*b*; Boardman, McKinney & Taylor, 1992; see also d'Hondt, 2005, 2015). The emergence of molecular techniques resulted in various phylogenetic trees which contradict each other in many aspects (Dick *et al*., 2000; Fuchs, Obst & Sundberg, 2009; Tsyganov‐Bodounov *et al*., 2009; Knight, Gordon & Lavery, 2011; Waeschenbach, Taylor & Littlewood, 2012). The classical view on the interrelationships among Cyclostomata also was challenged, suggesting a high level of homoplasies in this group (Waeschenbach *et al*., 2009).

**Figure 2:**
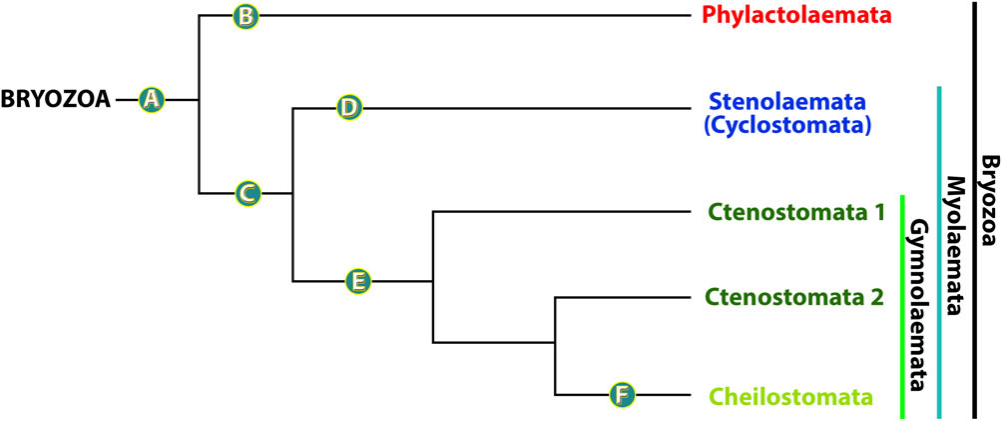
Interrelationships of the major taxa of Bryozoa, main topology redrawn after Waeschenbach *et al*. (2012). Branches A–F each represent a set of characters present in the particular clades (see Section II.1*a*–*g* for details).

As to the phylum Bryozoa in general, the most recent molecular analysis lends support for the Phylactolaemata being sister to the remaining Bryozoa and the Stenolaemata (Cyclostomata) being sister to Gymnolaemata (Waeschenbach *et al*., 2012; Fig. 2). Recently, the phylogeny of bryozoans based on molecular, skeletal and some soft tissue characters was reviewed by Taylor & Waeschenbach (2015). However, a large‐scale comparative analysis with the aim to identify distinct morphological features (apomorphies) that help to test the nodes obtained by the molecular trees and to reconstruct the evolution of the soft body characters within the phylum is still lacking. Herein, we plot morphological characters onto the currently most accepted bryozoan phylogenetic tree in order to identify ancestral and apomorphic characters. In addition, we draw conclusions on character evolution for the entire phylum.

## II. CHARACTER EVOLUTION

### (1) Character distribution among the major clades

Below we analyse the distribution of morphological characters, predominantly of soft tissues, throughout the major bryozoan clades. Larval or ontogenetic features are not studied sufficiently for a broad comparison to be included here. In addition, phylactolaemates and cyclostomes have derived reproductive patterns (see Section II.2*(f)*) that have little in common with gymnolaemates. The choice of characters is mainly based on reliable characters that can be distinctly allocated to the different clades on the tree. In the below descriptions, characters are listed according to the initial of the taxon name.

#### (a) *Bryozoa (Branch A in Fig. 2)*

##### (i) General characters

B1: *Coloniality*. Apart from very few solitary species that have secondarily acquired this lifestyle (see Schwaha, 2019*a*), bryozoans are the only animal phylum that is exclusively colonial. Colonies are composed of structurally and physiologically interconnected modules (zooids) (Ryland, 1970, 1976; McKinney & Jackson, 1989).

B2: *Zooidal budding*. Zooids are formed by iterative asexual budding (somatic morphogenesis) and are thus genetically identical (clones) to the founding zooid (ancestrula) of a colony (Boardman *et al*., 1983).

B3: *Autozooidal structure*. An individual feeding zooid (autozooid) is composed of the cystid (body wall consisting of a cuticular ectocyst that is often calcified, and cellular endocyst including epidermis, peritoneum and associated musculature as well as peripheral nervous system) and the polypide (the lophophore, i.e. the food‐gathering apparatus with ciliated tentacles, a U‐shaped digestive tract with the mouth within and the anus outside the lophophore crown, and central nervous system) (Mukai *et al*., 1997; Schwaha, 2019*b*).

B4: *Polypide formation*. In sexual and asexual development, the polypide is always formed from a two‐layered anlage/bud (Nielsen, 1971; Reed, 1991). Metamorphosis after larval settling is commonly catastrophic, and the two layers of the polypide anlage are formed either from delamination of blastemal cells (Nielsen, 1970) or their condensation at the apical pole (Zimmer & Woollacott, 1977*a*; Reed, 1991). During zooidal budding, the polypide is formed by invagination of the two‐layered cystid wall (Mukai *et al*., 1997). In the ctenostome *Hislopia malayensis* it was suggested that some parts of larval tissues may be incorporated into the polypide of the founding zooid (ancestrula) (Wood, 2008), but this requires confirmation.

B5: *Polypide retraction* via *body wall inversion*. In this defensive mechanism typical of all Bryozoa, the polypide is pulled into the protective cystid *via* inversion of the tentacle sheath (functionally an introvert) following contraction of the retractor muscles, commonly the most prominent muscular element (Mukai *et al*., 1997).

B6: *Polypide protrusion by body wall compression*. The lophophore is protruded from the zooidal aperture or orifice (orifice and aperture are often used synonymously, but strictly ‘orifice’ is soft‐bodied and ‘aperture’ skeletal; Schwaha, 2019*b*) and expands as a result of an increase in coelomic fluid pressure due to the compression of a flexible area of the body wall (Phylactolaemata, Gymnolaemata) or a membranous sac (Stenolaemata) (Hyman, 1959; Nielsen & Pedersen, 1979; Mukai *et al*., 1997).

B7: *Budding direction*. Budding of new zooids (and, thus, colonial growth) in Bryozoa occurs either on the oral or anal side of the maternal zooid (with respect to the position of the mouth and anus of the U‐shaped gut; see Fig. 1). Whereas almost all phylactolaemates show an oral growth direction, all other bryozoans have an anal growth direction in their colonial development (astogeny). Different budding directions of Phylactolaemata *versus* the remaining bryozoans was emphasized by Jebram (1973*b*). Other budding lophotrochozoans such as kamptozoans as well as phoronids produce their buds predominantly on the oral side (kamptozoan buds often occur laterally) (Du Bois‐Reymond Marcus, 1949; Jebram, 1973*b*, 1986*a*; Emschermann, 1995), which was used as an argument for considering the oral budding direction as a plesiomorphic state. It should be noted that species from early branches of all larger bryozoan clades also show lateral budding (see Schwaha, Hirose & Wanninger, 2016).

B8: *Timing of polypide formation*. The formation of the polypide preceeds the formation of the cystid in Phylactolaemata and Cyclostomata (Borg, 1926) and *vice versa* in Gymnolaemata (Reed, 1991; Mukai *et al*., 1997).

B9: *Funiculus*. The proximal end of the stomach (caecum) of the U‐shaped gut is connected with the body wall or a pore‐cell complex by a tubular peritoneal cord with a lumen inside. In sexual zooids the funicular cord is often associated with gonads supposedly providing nutrition for gametogenesis. It is supplemented by muscular elements in species of all three larger bryozoan clades (Mukai *et al*., 1997).

B10: *Serotonin‐like immunoreactive (lir) distribution in the nervous system*. The distribution of serotonin is restricted to the lophophore base – the cerebral ganglion, circum‐oral nerve ring and perikarya (Schwaha, Wood & Wanninger, 2011b; Schwaha & Wanninger, 2012, 2015; Shunkina *et al*., 2015; Gruhl & Schwaha, 2015; Temereva & Kosevich, 2016). Similar patterns are also apparent for other neuroactive compounds such as FMRFamide (Shunkina *et al*., 2015), but analyses are currently restricted to the Phylactolaemata.

##### (ii) *Lophophorate characters*

The phylogenetic relationships of Bryozoa to other lophotrochozoans remain controversial. Traditionally they were grouped with the phyla Phoronida and Brachiopoda as Tentaculata or Lophophorata, which, however, was rejected in most molecular phylogenies (e.g. Dunn *et al*., 2008; Hejnol *et al*., 2009; Mallat, Craig & Yoder, 2012; reviewed in Ostrovsky, 2013*a*). Three recent molecular studies indicated or supported a monophyletic Lophophorata (Nesnidal *et al*., 2013, 2014; Marlétaz *et al*., 2019), whereas others rejected this concept (Cannon *et al*., 2016; Kocot *et al*., 2017). Recent morphological data on the nervous system show detailed similarties between bryozoans and phoronids (Temereva & Tsitrin, 2015; Temereva & Kosevich, 2016; Temereva, 2017*b*). In particular, the adult nervous system of the phoronid *Phoronis ovalis* was considered a ‘link’ between phoronids and bryozoans (Temereva, 2017*a*). Concerning other putative outgroups, little support is given to any alternative interpretation. Morphologically, there is little evidence that would unite bryozoans with any other phylum. Depending on the prospective sister group, lophophorate‐like characters were either present in the last common ancestor of bryozoans and a phoronid–brachiopod clade, or evolved convergently in each of these clades (e.g. Kocot *et al*., 2017). These characters include:

L1: *Lophophore*. A ciliated tentacle crown supplied with a coelomic canal in each tentacle. In some brachiopods, most phoronids and phylactolaemate bryozoans the lophophore is principally horseshoe‐shaped with large arms extending in the anal direction. Particularly in brachiopods, but also in some phoronids, these arms or branches can be rather long and often coiled (e.g. James, 1997; Temereva & Malakhov, 2009). Each tentacle of the lophophore in all three taxa has two sets of longitudinal muscles (frontal and abfrontal) and three sets of cilia: lateral (in two bands), laterofrontal (two bands) and frontal (Hyman, 1959; Reed & Cloney, 1977; Temereva & Malakhov, 2009; Nielsen, 2012; Schwaha & Wanninger, 2012). All three phyla possess a so‐called ‘upstream’ food‐collecting mechanism (Nielsen, 1987; Mukai *et al*., 1997; Nielsen & Riisgård, 1998) with similarities in food particle retention and transport (Gilmour, 1978; Nielsen & Riisgård, 1998). In contrast to phoronids and brachiopods, bryozoans possess multiciliate cells (Nielsen, 2002), which in a monophyletic Lophophorata could represent an apomorphy of Bryozoa. Suspension feeders such as the Kamptozoa also possess multiciliate cells. Tentacles in both the Bryozoa and Kamptozoa have only a small number of cells (9–12) in cross section, whereas phoronids and brachiopods have several dozen (~40–80) (see Mukai *et al*., 1997; Nielsen & Jespersen, 1997). It can be concluded that multiciliation might be a result of smaller size and fewer cells in bryozoans and kamptozoans.

L2: *Coelom*. All lophophorates have one or two large coelomic cavities that consist of a trunk (visceral) and a lophophoral coelom. These two cavities are commonly fully separated in phoronids (Herrmann, 1997; Gruhl, Grobe & Bartolomaeus, 2005), mostly incompletely separated in brachiopods (Hyman, 1959), and may be confluent or separated in bryozoans (Gruhl, Wegener & Bartolomaeus, 2009; Shunatova & Tamberg, 2019). A so‐called epistome or preoral lobe (present in phoronids, brachiopods and phylactolaemate bryozoans) has been traditionally considered to contain a third, separate coelomic component, but its presence is ambiguously discussed in lophophorates (see Hyman, 1959; Lüter, 1996; Bartolomaeus, 2001; Gruhl, Grobe & Bartoloameus, 2005; Grobe, 2008; Temereva & Malakhov, 2011; Temereva & Tsitrin, 2015; Temereva, 2015; Santagata, 2015).

Lophophorates have previously been considered closely related to deuterostomes, sometimes even as a link between protostomes and deuterostomes (e.g. Hyman, 1959; Salvini‐Plawen, 1982; reviewed in Ostrovsky, 2013*a*). Consequently, a trimeric arrangement in basal deuterostomes (hemichordates, echinoderms) was postulated for lophophorates too with three coelomic cavities termed the proto‐, meso‐ and metacoel corresponding to the epistomial, lophophoral and visceral/trunk body regions (e.g. Hyman, 1959). However, because none of the lophophorates has any close relationship to deuterostomes and they are nested within the protostome Lophotrochozoa (e.g. Halanych *et al*., 1995; Dunn *et al*., 2008; Hejnol *et al*., 2009; Kocot *et al*., 2017), this old hypothesis has been rejected. It is possible that a lophophorate ancestor independently evolved three consecutive coelomic cavities, but this is rather unlikely since none of the other lophotrochozoans show a similar body plan.

Communication of the coelom with an external medium occurs *via* coelomopores in bryozoans. Terminal tentacle pores are probably present in all bryozoans and have been shown in non‐phylactolaemates to release sperm. A supraneural coelomopore leads to the lophophoral coelom in the Gymnolaemata and presumably in the Stenolaemata and in the former is used for insemination and zygote release. Phylactolaemates possess a so‐called vestibular pore which leads to the trunk coelom and is used for statoblast, coelomocyte and sperm release (Ostrovsky & Porter, 2011; Schwaha *et al*., 2016). By contrast, Phoronida and Brachiopoda possess so‐called mixonephridia, i.e. metanephridia that also function as gonoducts (Hyman, 1959; Herrmann, 1997). Possibly, the lack of a blood vascular system including podocytes and miniaturization of zooids had an influence on the reduction of the nephridial system in bryozoans.

#### (b) *Phylactolaemata (Branch B in Fig. 2)*

**Figure 3:**
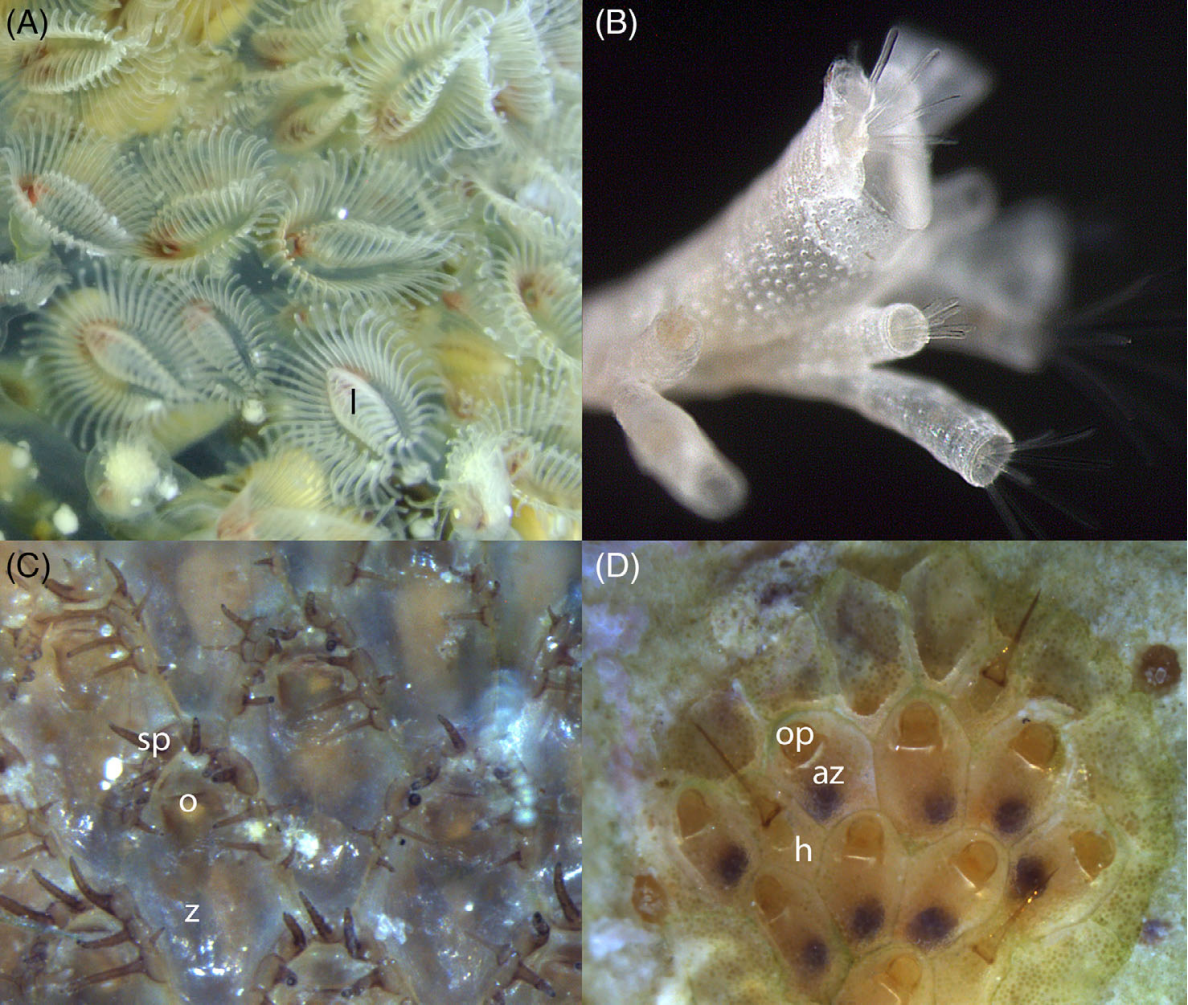
Representatives of major bryozoan clades. (A) Phylactolaemata: *Pectinatella magnifica* (showing horseshoe‐shaped tentacle crown). (B) Stenolaemata (Cyclostomata): *Crisiella producta* (courtesy of Olga Kotenko). (C) Gymnolaemata, ‘Ctenostomata’: *Flustrellidra hispida*. (D) Gymnolaemata, Cheilostomata: *Smittipora* sp. Abbreviations: az, autozooid; h, heterozooid (avicularium); l, lophophore; o, orifice; op, operculum; sp, spines; z, zooid.

P1: *Horseshoe‐shaped lophophore* (Fig. 3A). The tentacles of the lophophore are situated on two lophophoral arms that are elongated in the anal direction. The arms are secondarily reduced in the Fredericellidae but the horseshoe shape remains evident in specimens with retracted polypides and during ontogeny (Marcus, 1926*b*; Du Bois‐Reymond Marcus, 1946, 1953; T.F. Schwaha, personal observations). In addition, the nervous system shows greatly reduced ganglionic horns, i.e. the ganglionic extensions that reach into the lophophoral arms in the horseshoe‐shaped lophophore (Gruhl & Bartoloameus, 2008; Shunkina *et al*., 2015). The number of tentacles varies from 24 to more than 100 in species with horseshoe‐shaped lophophores and 20–23 in fredericellids (Wood, 2014; Shunkina *et al*., 2015). Depending on the outgroup for bryozoans, it is unclear whether the horseshoe‐shaped lophophore is an apomorphic character for Phylactolaemata or ancestral for all bryozoans (see also Section II.(3)).

**Figure 4:**
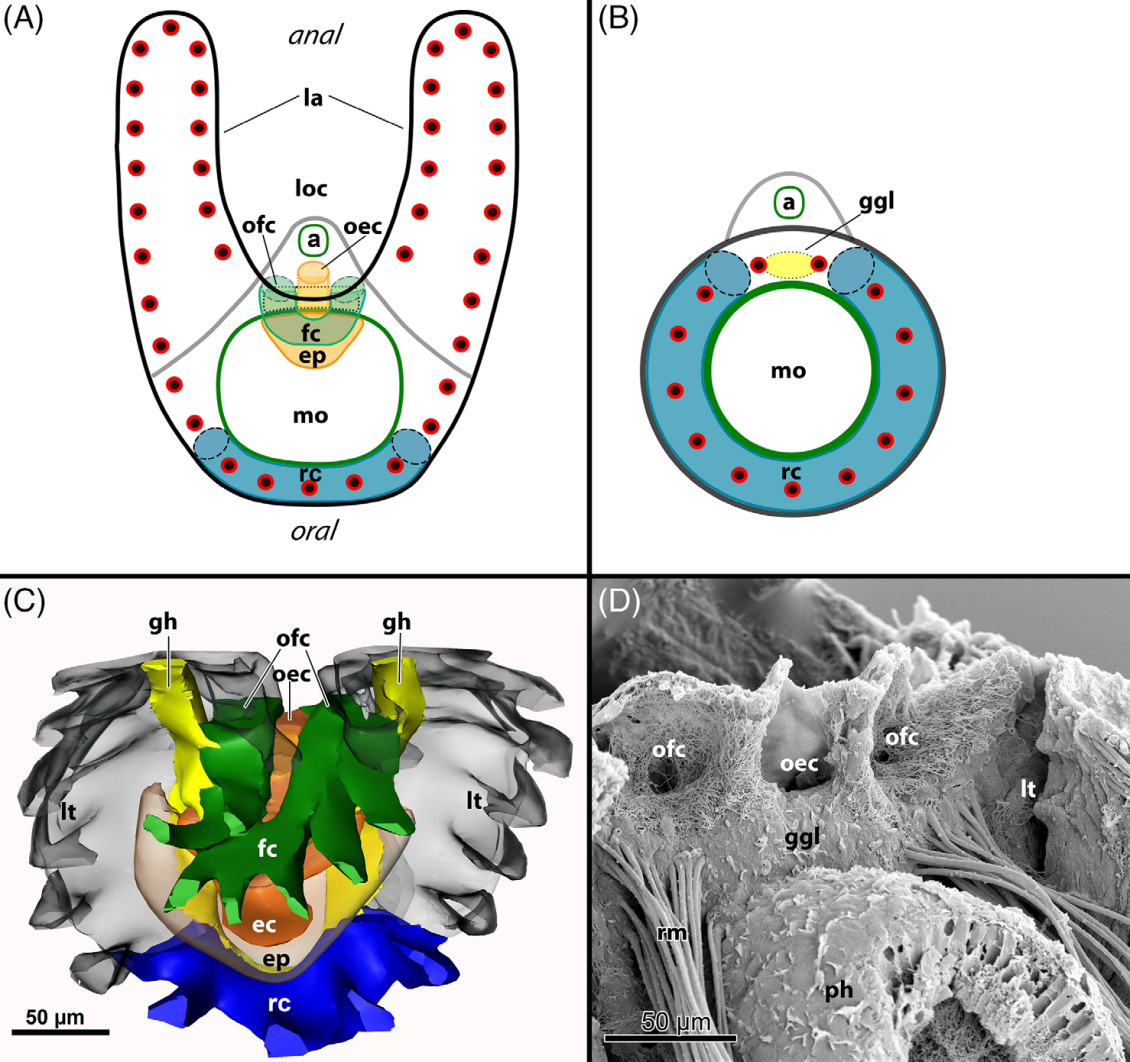
Coelomic system of bryozoans with a focus on the more complex situation in Phylactolaemata. (A, B) Schematic drawings of the lophophore base in a phylactolaemate (A) and a myolaemate (B) bryozoan. Dimensions and proportions, especially of the mouth opening, are exaggerated for clarity. Tentacles are displayed as red circles with black centres. (A) Phylactolaemates have a complex tentacle crown with large lophophoral arms. They possess a short ring canal supplying the oral row of tentacles whereas the tentacles of the inner lophophoral concavity are supplied by the forked canal. The forked canal sits on a protrusion of the coelomic cavity supplying the epistome which itself protrudes medially of the ganglion into the direction of the mouth opening. Tentacles emanating from the forked canal have been omitted in the drawing. See also C and D. (B) General condition of the lophophoral base of a myolaemate bryozoan. The tentacle crown is circular and only a ring canal is present which supplies the tentacles. This canal is open at the site of the ganglion (dashed line) which is again situated on the anal side of the polypide. Homology of the ‘ring canals’ in phylactolaemate and myolaemate bryozoans is unknown. (C) Three‐dimensional reconstrucion of the lophophoral base of the phylactolaemate *Cristatella mucedo* (serial semithin sections) showing the different coelomic compartments described in A. (D) Scanning electron micrograph of a broken zooid of *Cristatella mucedo*, viewed from the anal side of the pharynx. The opening of the epistomial cavity (located above the ganglion) is in direct connection with the remaining visceral coelomic cavity. On both lateral sides of the epistomial opening, heavily ciliated openings of the forked canal are situated. Abbreviations: a, anus; ec, epistomial coelom; ep, epistome; fc, forked canal; ggl, ganglion; gh, ganglionic horns; la, lophophoral arms; loc, lophophoral concavity; lt, lateral tentacles; mo, mouth opening; oec, opening of the epistomial coelom; ofc, opening of the forked canal; ph, pharynx; rc, ring canal; rm, retractor muscle.

P2: *Coelomic canals of the lophophore*. In Phylactolaemata the coelom supplying the tentacles of the lophophore is largely unrestricted towards the remaining trunk or visceral cavity. Tentacles at the lophophoral base are supplied by one of two canals: the ring canal which is connected to a few oral tentacles, and the forked canal which supplies the innermost set of tentacles in the inner lophophoral concavity of the horseshoe‐shaped tentacle crown (see Fig. 4A, C; see Gruhl *et al*., 2009; Schwaha *et al*., 2011a; Shunatova & Tamberg, 2019). It is not yet clear whether this arrangement is ancestral or evolved independently within Phylactolaemata, which are the only bryozoans to show this condition. A ring canal is present in non‐phylactolaemate bryozoans (Fig. 4B, and Section II.1*(c)*, M4), but is of uncertain homology with the ring canal in phylactolaemates.

P3: *Statoblasts*. These are dormant stages (buds) with a protective capsule for overwintering and dispersal. There are two different types – sessoblasts and floatoblasts – both of which develop inside the funiculus (Wood & Okamura, 2005; Wood, 2014). Sessoblasts are attached to the substrate whereas floatoblasts have an inflated annulus and are buoyant.

**Figure 5:**
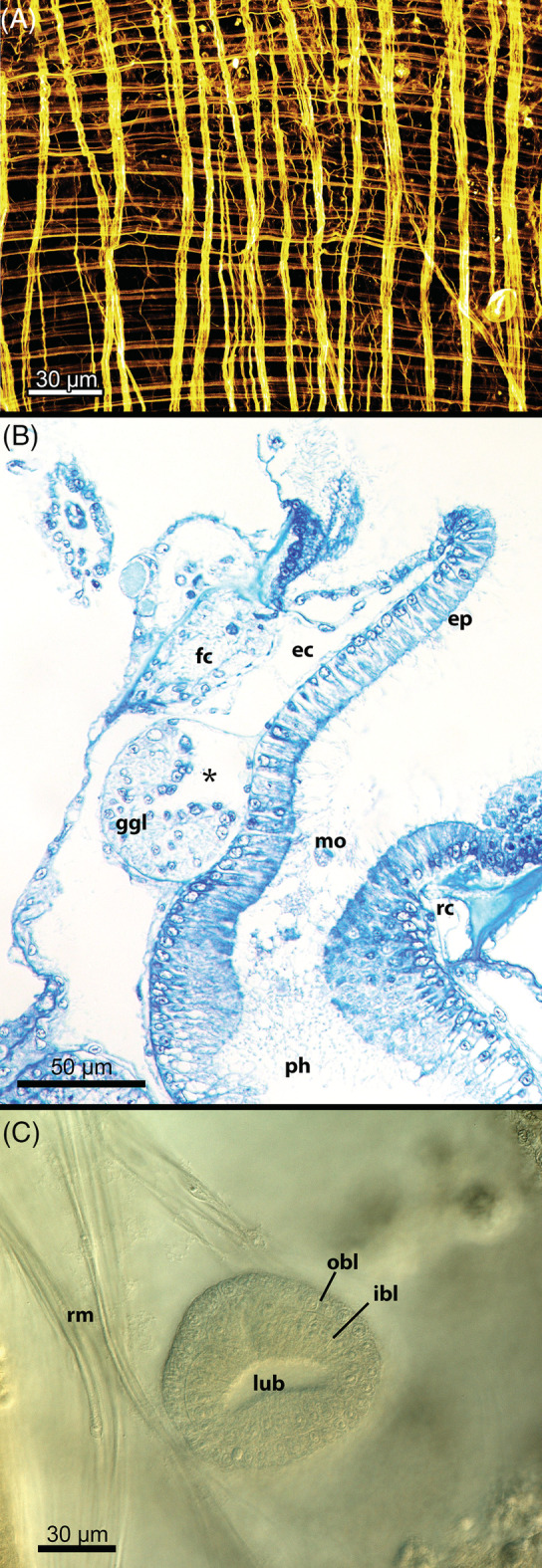
Morphological aspects of Phylactolaemata. (A) Regular arrangement of orthogonally oriented longitudinal and circular musculature of the body wall/endocyst of *Hyalinella punctata*. Confocal laser scanning micrograph with staining for f‐actin. (B) Ganglion adjacent to the pharyngeal epithelium with its central cavity (asterisk) in *Cristatella mucedo* (semithin sections, brightfield). (C) Polypide bud of *Cristatella mucedo* showing the two budding layers (differential interference contrast). Abbreviations: ec, epistome coelom; ep, epistome; fc, forked canal; ggl, ganglion; ibl, inner layer of the polypide bud; lub, lumen of the early bud; mo, mouth opening; obl, outer layer of the polypide bud; ph, pharynx; rc, ring canal; rm, retractor muscle.

P4: *Body wall musculature* (Fig. 5A). The phylactolaemate endocyst (cellular part of the body wall) carries a regular mesh of circular and longitudinal muscle fibres similar to the ‘Hautmuskelschlauch’ (muscular tube) of worm‐shaped organisms (Marcus, 1934; Schwaha & Wanninger, 2012; Gawin, Wanninger & Schwaha, 2017). This might represent an ancestral character of the last common bryozoan ancestor.

**Figure 6:**
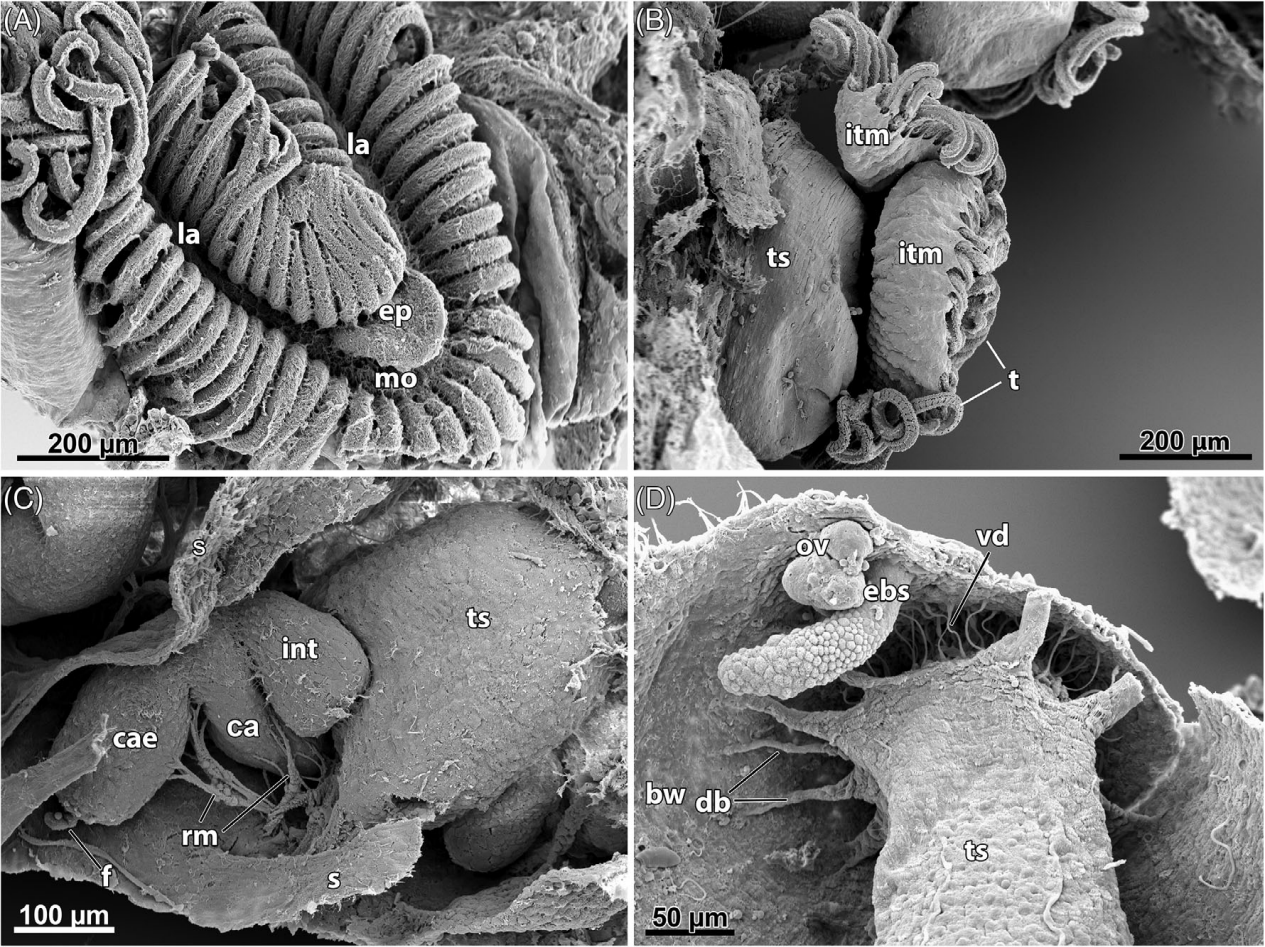
Scanning electron micrographs of phylactolaemate bryozoans. (A–C) *Cristatella mucedo*. (D) *Hyalinella punctata*. (A) View of the lophophore showing the horseshoe‐shaped arrangement of the tentacles and the epistome above the mouth opening. (B) Lateral view of two zooids with protruded lophophores showing the intertentacular membrane on the outer (abfrontal) side of the tentacle crown. (C) Dissected zooid with retracted polypide showing the tentacle sheath, digestive tract and retractor muscle attached to several parts of the gut. (D) Dissected zooid with retracted polypide showing the arrangement of the apertural muscle system (vestibular dilatators and the duplicature bands), ovary and embryo sac. Abbreviations: bw, body wall; ca, cardia; cae, caecum; db, duplicature bands; ebs, embryo sac; ep, epistome; f, funiculus; int, intestine; itm, intertentacular membrane; la, lophophoral arm; mo, mouth opening; ov, ovary; rm, retractor muscle; s, interzooidal septum; t, tentacles; ts, tentacle sheath; vd, vestibular dilatators.

P5: *Intertentacular membrane* (Fig. 6B). This is a thin epidermal duplicature between the tentacle bases of the lophophore. There is some variation in how this membrane spans between adjacent tentacles, which might be taxon‐specfic (see Braem, 1890). A distinct intertentacular membrane is lacking in non‐phylactolaemates. The distribution of specific neuroactive compounds implies that the intertentacular pits of Gymnolaemata and intertentacular bases of Cyclostomata (see Section II.1*(e)*, G6) could be homologous structures (see Schwaha, 2019*b*).

P6: *Six tentacle nerves*. Phylactolaemates show a set of six distinct tentacle nerves, three on the frontal (i.e. the side facing the mouth opening), and three on the abfrontal side (Shunkina *et al*., 2015, Ambros, Wanninger & Schwaha, 2018). Multiple thin frontal nerves were reported in the lophopodid *Asajirella gelatinosa* (Mukai *et al*., 1997) and also in other phylactolaemates (Tamberg & Shunatova, 2017) although it was not clear in which lophophoral region the latter were identified. It is likely that the analysed sections were from the area of the multiple frontal neurite bundle roots (see Section II.2*(c)*) rather than from distinct tentacles.

P7: *Duplicate polypide buds*. During asexual astogeny, the two‐layered early buds consist of the primordia of two developing consecutive polypides (eventually zooids) that separate after initial bud formation (Nitsche, 1875; Braem, 1890; Schwaha & Wood, 2011; Schwaha *et al*., 2011a).

P8: *Epistome* (Fig. 6A). This is a ciliated flap above the mouth supported by a coelomic extension of the trunk coelom, not a separate coelomic cavity (Fig. 4A, C). This structure is present in most phylactolaemates, but was reported to be absent in the lophopodid *Lophopus crystallinus* (Gruhl *et al*., 2009), contrary to previous reports (Marcus, 1934). A recent reinvestigation of this species showed that an epistome was present, as in all other phylactolaemates (Schwaha, 2018).

P9: *Hollow ganglion* (Fig. 5B). In all bryozoans the polypide is formed from an early bud consisting of two epithelial layers – an inner and an outer budding layer (e.g. Reed, 1991; Schwaha *et al*., 2011a; Schwaha & Wood, 2011). The cerebral ganglion is formed as an invagination of the inner budding layer in the area of the prospective pharyngeal epithelium which then closes to form a hollow ganglionic vesicle. The enclosed lumen remains present in phylactolaemates through zooidal life (Gruhl & Bartoloameus, 2008, Schwaha *et al*., 2011a; Shunkina *et al*., 2015) whereas almost all non‐phylactolaemates lack this lumen in functional polypides. Recently, a tiny ganglionic lumen was found to persist in two ctenostome species (Weber, Wanninger & Schwaha, 2014; Temereva & Kosevich, 2016). Since this lumen is so small, it may have been overlooked in other gymnolaemate species and consequently might not be a true apomorphy of Phylactolaemata. However, it should be noted that in cross section the cerebral ganglion in phylactolaemates always has a crescent‐shaped appearance, with tissue concentrated at the anal side whereas at the oral side it is developed as a thin membrane (Fig. 5B).

P10: *Embryonic brooding accompanied by matrotrophy*. Incubation of the growing embryo occurs inside an internal brood sac formed from an invagination of the body wall. It is supported by extraembryonic nourishment, presumably histotrophic and placental (Braem, 1890, 1897, 1908; Davenport, 1891; Mukai, 1982; Ostrovsky *et al*., 2016). Since the condition in early‐branching Stephanellidae is unknown, it is not clear whether this feature evolved at the base of Phylactolaemata or within the clade.

P11: *Mantle larva*. Most phylactolaemate larvae are commonly regarded as short‐lived swimming colonies (Mukai, 1982; Reed, 1991). Due to heterochronic shifts, adult structures, i.e. differentiated polypide(s) with bud(s), are formed during embryogenesis. Nonetheless, the presence of a ciliated larval hull or mantle justifies the morphological status of a larva. Also, the nervous system of the larval hull does not show any interconnection to the adult structures (Gruhl, 2010; Schwaha *et al*., 2015). So far, this larval type has been found in five out of six phylactolaemate families (Plumatellidae, Fredericellidae, Cristatellidae, Pectinatellidae and Lophopodidae) (Allman, 1856; Mukai, 1982) with the larva of Stephanellidae unknown. Recent molecular analyses placed Stephanellidae either as sister to all remaining phylactolaemates or as an early offshoot within the Lophopodidae (Hirose, Dick & Mawatari, 2008). With the identity of the stephanellid larva remaining unknown, it is difficult to assess whether the mantle larva of phylactolaemates represents an apomorphic feature or has evolved within the group.

P12: *Sperm type*. Phylactolaemate sperm show a distinct morphology compared to other bryozoans. They have an acrosome, the head region is drop‐shaped and the midpiece region is surrounded by numerous mitochondria. The latter is considered typical for a modified (i.e. internal) mode of fertilization (see Franzén, 1977; Lützen, Jespersen & Nielsen, 2009).

P13: *Radial symmetry in the apertural area* (Fig. 6D). The aperture is the area where retracted polypides emerge from the cystid. It includes the external opening, the orifice, where the body wall is invaginated to form the vestibular wall which is continuous with the thin wall of the tentacle sheath. The terms orifice and aperture are often used synonomously: orifice is generally used for all bryozoans whereas aperture is more often applied to the skeletal openings of Stenolaemata. In most cases, aperture and orifice refer to the same structure. This general area is here referred to as the ‘apertural area’ (see Schwaha, 2019*b*).

A distinct area between the vestibular wall and the tentacle sheath is termed the diaphragm and contains a strong sphincter which in retracted zooids closes the entrance of the apertural area to the tentacle sheath. Several other muscle bundles are associated with the apertural area: separate muscle fibres come from the body wall and insert in the proximal area of the vestibular wall (vestibular dilatators, parieto‐diaphragmaticus, parieto‐vestibularis) and muscular peritoneal bands that insert at the tentacle sheath or in the area of the diaphragm (duplicature bands and parieto‐vaginal bands; see Schwaha, 2019*b*). In Phylactolaemata these are arranged radially (Schwaha *et al*., 2011a), which is probably the ancestral state for bryozoans since the topologically similar attachment organ of Cyclostomata sometimes shows radial symmetry. This needs to be assessed in more detail (see Schwaha *et al*., 2011b).

P14: *Funiculus with basal lamina*. The peritoneal cord (funiculus) contains a central lumen that in Phylactolaemata is underlain by a basal lamina (true epithelial organization). This is considered to be a distinct condition compared with other Bryozoa (Carle & Ruppert, 1983).

#### (c) *Myolaemata (new clade) (branch C in Fig. 2)*

**Figure 7:**
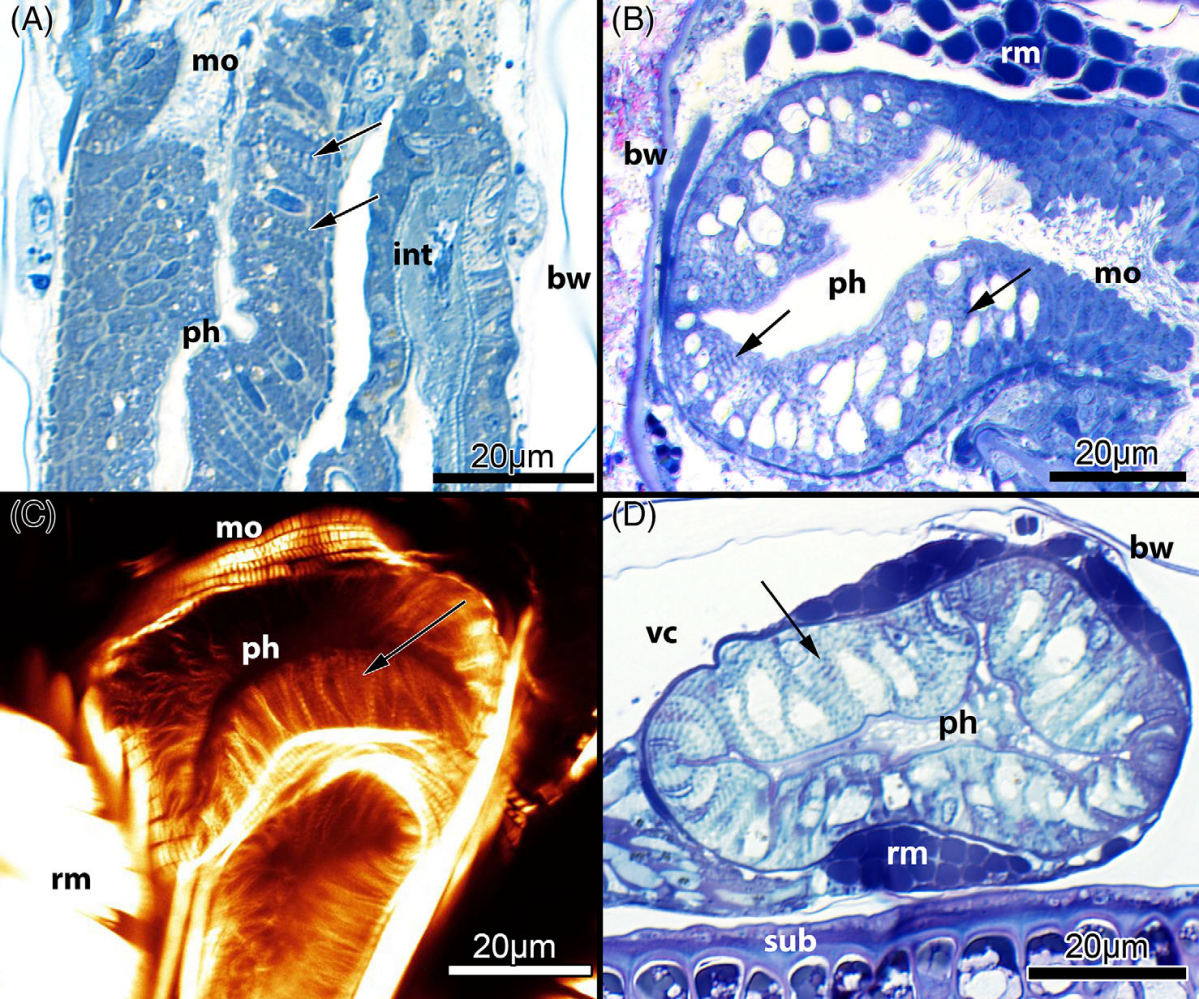
Pharyngeal anatomy of Myolaemata. (A) Pharynx of the cyclostome *Crisia* sp. (longitudinal semithin section). (B) Pharynx of the ctenostome *Arachnidium fibrosum* (oblique semithin section). (C) Pharynx of the cheilostome *Cellaria fistulosa*. Optical section, confocal laser scanning micrograph with staining for f‐actin. (D) Pharynx of the cheilostome *Celleporella hyalina* (oblique semithin section). Arrows in A–D point to the striated myofibrils of the pharyngeal epithelium. Abbreviations: bw, body wall; int, intestine; mo, mouth opening; ph, pharynx; rm, retractor muscle; sub, substrate; vc, visceral cavity.

In current molecular phylogenies Stenolaemata (Cyclostomata) and Gymnolaemata are sister taxa, which we define here as a new clade Myolaemata. Myolaemata is sister to Phylactolaemata (Fig. 2). The term Myolaemata is derived from the Greek *myo* meaning muscular and *laimos* meaning throat. Distinct morphological apomorphies that unite these two clades are as follows:

M1: *Myoepithelial pharynx* (Fig. 7). In Phylactolaemata the pharynx is surrounded by peritoneally derived ring musculature for peristaltic movements. In both Cyclostomata and Gymnolaemata, the epithelial cells of the pharynx form a myoepithelium with cross‐striated contractile fibres associated with the lateral membranes of these cells. Their sarcomeres are arranged perpendicularly to the longitudinal axis of the pharynx, causing shortening of the pharyngeal cells along the basoapical axis during contraction, and rapid expansion of the pharyngeal cavity resulting in suction. The triradial shape of the pharyngeal lumen additionally allows the myoepithelium to enlarge the gut volume and, as a result, its suction force (Marcus, 1939; Braem, 1940*a*; Matricon, 1973; Gordon, 1975*b*; Nielsen, 2013). Consequently, most bryozoans are not merely suspension/filter‐feeders, but also employ suction feeding (Borg, 1926; Shunatova & Ostrovsky, 2001; Ostrovsky, Shunatova & Antipenko, 2002). Activity of the pharyngeal suction pump is accompanied by contraction of a series of radially traversing buccal dilatators at the lophophoral base [Borg (1926) for Cyclostomata; Gordon (1974) and Schwaha *et al*. (2011b) for Gymnolaemata; (T. F. Schwaha, personal observations) for both taxa].

M2: *Circular lophophore*. Tentacle bases are always arranged in a circle. The number of tentacles generally ranges from 8 to 20 (Mukai *et al*., 1997). It is unknown whether the ancestral bryozoan condition is a circular or a horseshoe‐shaped lophophore, thus leaving the question of apomorphy *versus* plesiomorphy for this character unanswered.

M3: *Pylorus with cilia*. The cells of the pyloric area in the gut of both Cyclostomata and Gymnolaemata bear cilia that are lacking in phylactolaemates (Silén, 1944*a*). The significance of this and of differences in the muscular system are discussed below in Section II.2*b*.

M4: *Lophophoral coelomic cavity in the form of either an open or closed ring canal*. The coelomic cavity at the lophophoral base supplying the tentacles is smaller than in phylactolaemates, but supplies almost all tentacles of the lophophore. This cavity is completely separated from the visceral coelom by a circumpharyngeal dissepiment in cyclostomes, but remains open with two openings of variable size at the anal side of the pharynx in the Gymnolaemata (Shunatova & Tamberg, 2019) (see also Fig. 4B). The term ‘ring canal’ has been used previously for this cavity (e.g. Borg, 1926), but its homology to the phylactolaemate ring canal is questionable. The buccal dilatator muscles radially traverse this ring canal [Borg (1926) for Cyclostomata, Schwaha & Wood (2011) for Ctenostomata, and Gordon (1974) for Cheilostomata].

M5: *Anal growth direction*. In both Cyclostomata and Gymnolaemata, zooidal buds are formed on the anal side of the maternal zooid that defines the direction of colony growth (Du Bois‐Reymond Marcus, 1949; Jebram, 1973*b*, 1986*a*). Budding patterns are discussed in more detail below in Section II.(3).

M6: *Zooidal polymorphism*. There is morphofunctional specialization of zooids in a colony affecting either the cystids or polypides, or both. Feeding zooids are termed autozooids whereas zooids with other functions are termed autozooidal (with a functional polypide) or heterozooidal (polypide reduced) polymorphs (Silén, 1977; Cheetham & Cook, 1983; Lidgard *et al*., 2012; Schack, Gordon & Ryan, 2019).

M7: *Polypide recycling*. Polypides degenerate inside zooids in a regular fashion to form residual ‘brown bodies’. This phenomenon may be related to accumulation of excretory waste products in polypide cells (Gordon, 1977). The polypide can regenerate *via* internal budding processes. Brown bodies can be incorporated into the lumen of the developing stomach of buds (finally being defaecated), but are commonly retained inside the zooidal coelom (Harmer, 1891; A. N. Ostrovsky, personal observations). In the latter case, the number of brown bodies indicates the number of recycling events. Polypides do not regenerate in Phylactolaemata (Mukai *et al*., 1997).

M8: *Sperm morphology*. Similar elongated sperm heads are present in both groups, with 2–4 mitochondria in the midpiece. An acrosome is described for Cyclostomata, but is absent in most gymnolaemates (Franzén, 1977, 1987; Mukai *et al*., 1997).

*Additional remarks on Myolaemata*. Additional apomorphies of the sister‐group relationships of Stenolaemata and Gymnolaemata are the absence of an epistome, a funiculus without a basal lamina, and complete body walls (with communication pores plugged by cells) separating neighbouring zooidal cavities (Taylor & Waeschenbach, 2015). The absence of characters may be of phylogenetic importance (see e.g. Bleidorn, 2007), however, their significance remains poorly understood in bryozoans.

The recognition of the new clade Myolaemata predominantly relies on soft body morphology. Stenolaemata includes numerous extinct taxa and it is not possible to assess whether any of these extinct groups – including Palaeozoic cyclostomes – possessed these anatomical features.

#### (d) *Stenolaemata (Cyclostomata) (branch D in Fig. 2)*

S1: *Calcified skeleton* (Fig. 3B). Many stenolaemates and all cyclostomes possess tubular calcified cystids. Cumulative evidence indicates that the ancestral forms were non‐calcified and that their calcification evolved independently from the calcification of the gymnolaemate Cheilostomata (e.g. Todd, 2000; Ernst & Schäfer, 2006; Taylor, 2008; Taylor, Lombardi & Cocito, 2015b).

S2: *Membranous sac*. In cyclostome bryozoans the peritoneal layer is detached from the epidermal layer of the body wall, thus forming an internal sac that contains the original coelomic cavity (endosaccal space) separate from the space between the epidermis and detached peritoneum (the exosaccal cavity) (Borg, 1926; Nielsen & Pedersen, 1979; Mukai *et al*., 1997; Boardman, 1998; U. A. Nekliudova, T. F. Schwaha & A. N. Ostrovsky, unpublished data). Due to their extensive calcification, cyclostomes lack compressible areas that effectuate polypide eversion. Compression of the fluid is achieved by annular ring muscles located in the wall of the membranous sac (Nielsen & Pedersen, 1979; Taylor, 1981).

S3: *Gonozooids*. Specialized voluminous polymorphic zooids are used for embryonic incubation. They are non‐feeding, and are ontogenetically derived from ordinary autozooids that possess an ovary. Gonozooids are only unknown in the family Cinctiporidae which may incubate their embryos inside voluminous zooids (Boardman, Mckinney & Taylor, 1992; Schwaha *et al*., 2018). The incubation chamber of the family Lichenoporidae comprises two or several female zooids (Borg, 1926).

S4: *Polyembryony*. As far as is known, all cyclostomes possess polyembryony, i.e. a fertilized egg gives rise to the primary embryo that buds more than a hundred larvae (Harmer, 1893, 1896, 1898; Robertson, 1903; Borg, 1926; Jenkins *et al*., 2017; U. A. Nekliudova, T. F. Schwaha & A. N. Ostrovsky, unpublished data). The situation in Cinctiporidae remains unknown.

S5: *Matrotrophic viviparity*. Embryonic multiplication and growth in the maternal coelom are accompanied by nutritional provisioning *via* a placental analogue (Harmer, 1893, 1896, 1898; Borg, 1926; Ostrovsky *et al*., 2016; U. A. Nekliudova, T. F. Schwaha & A. N. Ostrovsky, unpublished data). Like the above‐mentioned reproductive characters, this feature is currently unknown in Cinctiporidae but is assumed to be present (Schwaha *et al*., 2018).

S6: *Dome‐shaped protoecium*. Recent and fossil cyclostomes commonly possess a dome‐shaped (semispherical) protoecium, i.e. the basal part of the founding zooid/ancestrula formed from the settled and metamorphosed larva (Taylor, Hara & Jasionowski, 2006; Taylor *et al*., 2015a).

S7: *Absence of mediofrontal cilia*. Tentacles in Phylactolaemata and Gymnolaemata possess a mediofrontal row of ciliary cells. By contrast, these cells are lacking cilia in Cyclostomata. There are indications that cyclostomes have lost the mediofrontal cilia and instead use tentacle flicking for particle transport towards the mouth (Nielsen & Riisgård, 1998). Alternatively, retained particles might be transported towards the downwardly directed core current by flicking of the normally stiff laterofrontal cilia or by local beat reversals of the lateral cilia as suggested for Gymnolaemata (Riisgård, Okamura & Funch, 2010). In at least three different cyclostome clades, the frontal surface secretes ‘mucus’‐like droplets that implies feeding by mucus entrapment in these bryozoans (Schwaha, 2019*b*).

#### (e) *Gymnolaemata (branch E in Fig. 2)*

**Figure 8:**
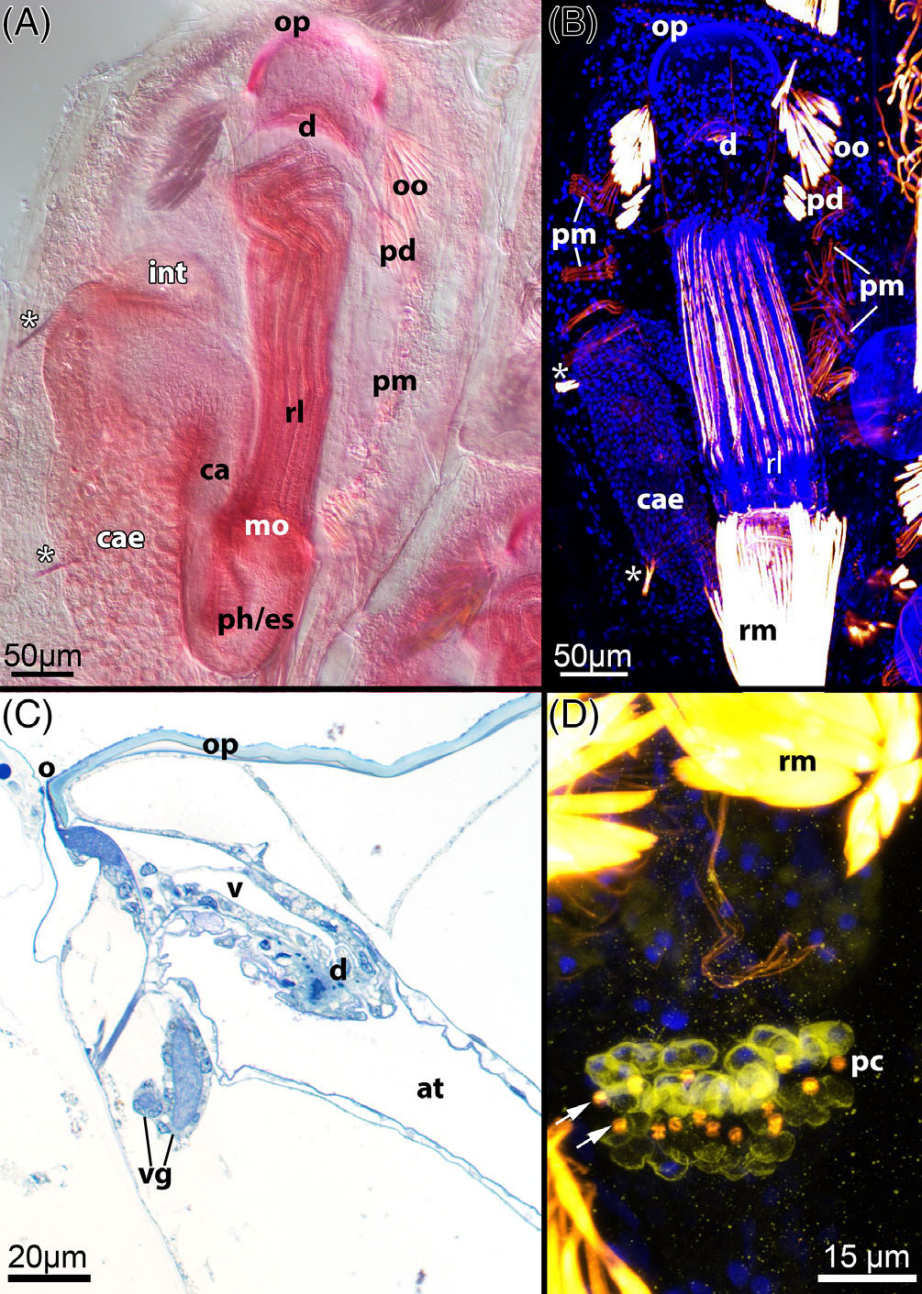
Morphological characters of the Gymnolaemata, Cheilostomata. (A) Zooid with retracted polypide of *Electra* sp. Asterisks mark the funicular cords/caecal ligaments attached to the caecum. Whole mount, decalcified and stained with boraxcarmine. (B) Zooid with retracted polypide of *Electra posidonia*. Asterisks mark the funicular cords/caecal ligaments attached to the caecum. Maximum projection of confocal laser scanning microscopy (CLSM) stack with staining for f‐actin (bright areas) and cell nuclei (blue). (C) Apertural region of *Calyptotheca hastingsae* showing vestibulum, diaphragm and vestibular gland (longitudinal semithin section). (D) Multiporous pore complex in the body wall between two neighbouring zooids of *Cellaria fistulosa*. Arrows point to the cincture cells of the pore‐cell complexes. Maximum projection of CLSM stack with staining for serotonin‐like immunoreactivity (yellow), f‐actin enrichment (bright areas, also indicated by arrows) and nuclei (blue). Abbreviations: at, atrium; ca, cardia; cae, caecum; d, diaphragm; es, esophagus; int, intestine; mo, mouth opening; o, orifice; oo, operculum occlusor; op, operculum; pc, pore‐cell complex; pd, parieto‐diaphragmatic muscle; ph, pharynx; pm, parietal musculature; rl, retracted lophophore; rm, retractor muscle; v, vestibulum; vg, vestibular gland.

G1: *Parietal musculature* (Fig. 8A, B). The muscles of the hydrostatic mechanism providing tentacle protrusion are present as a series of transversely oriented bundles originating from the body wall on the lateral or basal side of each zooid and attaching to the frontal side (Mukai *et al*., 1997). Number, size and attachment loci of the bundles vary among gymnolaemate taxa and depend on zooid morphology. These muscles are not shared with stenolaemates in contrast to previous statements (Taylor, 1981; Taylor & Waeschenbach, 2015), since stenolaemates have annular muscles in the membranous sac. Both parietal and annular muscles are likely to have originated from the circular muscle layer of the ancestral body wall that was modified during its calcification (Cyclostomata, Cheilostomata) or during reduction (Gymnolaemata) or displacement (Cyclostomata) of the peritoneal lining (see Sections II.1*d* and II.2*a*).

G2: *Pore‐cell complexes (rosettes)*. Neighbouring zooids are interconnected *via* pores in zooidal walls plugged by special pore‐cell complexes (see Gordon, 1975*c*; Bobin, 1977; Mukai *et al*., 1997). They typically consist of three types of cells: so‐called ‘special cells’, cincture cells and limiting cells. The coelomic cavity of each gymnolaemate zooid is thus individually separated in contrast to the situation in Phylactolaemata where free interchange of coelomic fluid between zooids occurs (Mukai *et al*., 1997). Both open and closed interzooidal communication pores are present in Cyclostomata (Carle & Ruppert, 1983; Nielsen & Pedersen, 1979; U. A. Nekliudova, T. F. Schwaha & A. N. Ostrovsky, personal observations).

**Figure 9:**
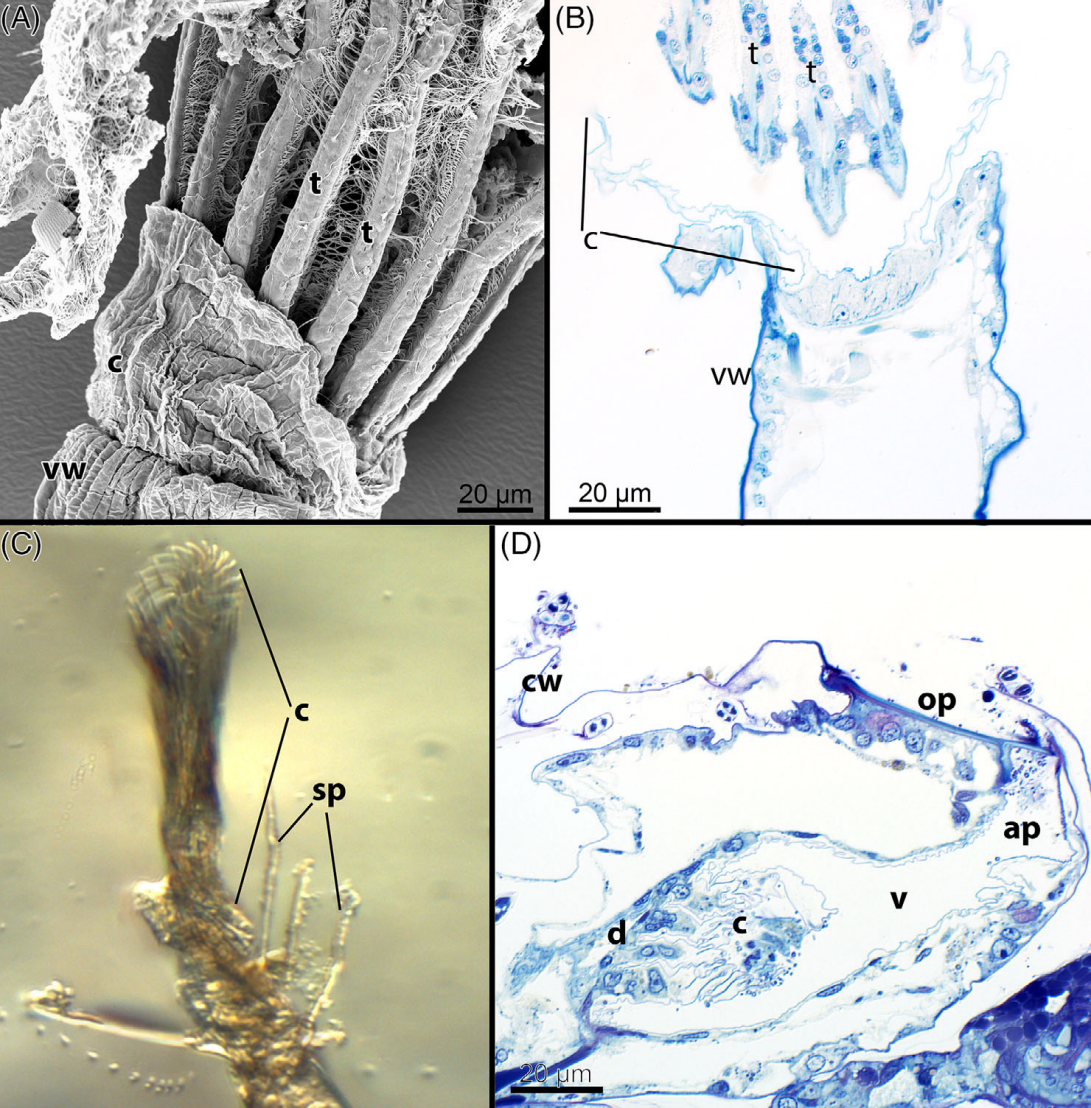
The collar of the Gymnolaemata. (A, B) Partially protruded lophophore of the ctenostome *Paludicella articulata* showing the acellular collar. A, scanning electron micrograph; B, longitudinal semithin section. (C) Partially exposed giant collar of the ctenostome *Aeverrillia setigera*. This constitutes the longest, setigerous collar found in any species and in a contracted state twirls into helicoidal lamellae. (D) Distal region of an autozooid with retracted tentacle crown of the cheilostome *Celleporella hyalina* (longitudinal semithin section). Abbreviations: ap, aperture; c, collar; cw, cystid wall; d, diaphragm; op, operculum; sp, spines; t, tentacle; v, vestibulum; vw, vestibular wall.

G3: *Collar* (Fig. 9). The collar represents an acellular protrusion at the proximal side of the vestibular wall. It varies in its morphology (see McKinney & Dewel, 2002), sometimes being supported by regular, stiff cuticular rods giving a serrated/pleated comb‐like appearance. Although it has been traditionally used as a diagnostic character for the ‘Ctenostomata’, it has also been detected in several cheilostomes [see Banta, Perez & Santagata (1995); Fig. 9D; also shown in Ostrovsky (1998)] and appears to be a shared character that was lost independently multiple times among gymnolaemates, including in a few ctenostomes: e.g. supposedly in the alcyonidiid genus *Elzerina* (Banta, 1975) and apparently is absent in the genus *Panolicella* (Jebram, 1985). The collar serves as a protective structure, blocking the orifice when the polypide is retracted. It has been noted previously that some ctenostome genera (e.g. *Elzerina* sp.) that have a reduced collar possess operculum‐like closing structures instead (see Section II.1*g*), and thus its reduction appears correlated with the introduction of new defensive apparatus to close the zooidal aperture/orifice (Banta, 1975). However, the ctenostome *Panolicella nutans* has upright zooidal tubes with no collar but apparently without a substitute defensive structure (Jebram, 1985; Vieira, Migotto & Winston, 2014). By contrast, both protective structures can occur in some cheilostomes (Banta *et al*., 1995; Ostrovsky, 1998).

G4: *Fourfold symmetry in the apertural area*. The basal configuration of Gymnolaemata includes three sets of apertural muscles. There is a general pattern of four parieto‐vestibular and parieto‐diaphragmatic muscles as well as four duplicature bands (sometimes previously referred to as ‘parieto‐vaginal bands’; Schwaha *et al*., 2011b, Schwaha & Wanninger, 2012, 2018; Schwaha, 2019*b*). In Cheilostomata and Ctenostomata with box‐shaped zooids, the parieto‐diaphragmatic and parieto‐vestibular mucles are reduced to a single lateral pair. The original fourfold symmetry is still reflected in the number of duplicature bands (Schwaha, 2019*b*; see also Lutaud, 1983). Some species have additional duplicature bands, for example, there are eight in the cheilostome *Bugulina simplex* [see Calvet (1900), as *Bugula sabatieri*] and six in *Pherusella* cf. *brevituba* and *Sundanella* sp. (T. F. Schwaha, personal observations). Since most analysed ctenostomes show four or fewer, it appears likely that the observed duplication is a secondary condition among a few cheilostomes.

G5: *Zooidal budding*. The cystid is produced first whereas the polypide forms later during ontogeny. An additional feature is that the polypide buds experience a 90° shift in their original orientation during early development (see Borg, 1926; Lutaud, 1983; Schwaha & Wood, 2011). This means that early‐forming organs like the tentacles and lophophore are initially oriented perpendicular to the frontal zooidal wall from which the bud originates. During development the 90° shift yields an orthogonal arrangement of these organs relative to the basal‐frontal axis, with the lophophore then facing the distal growth edge.

**Figure 10:**
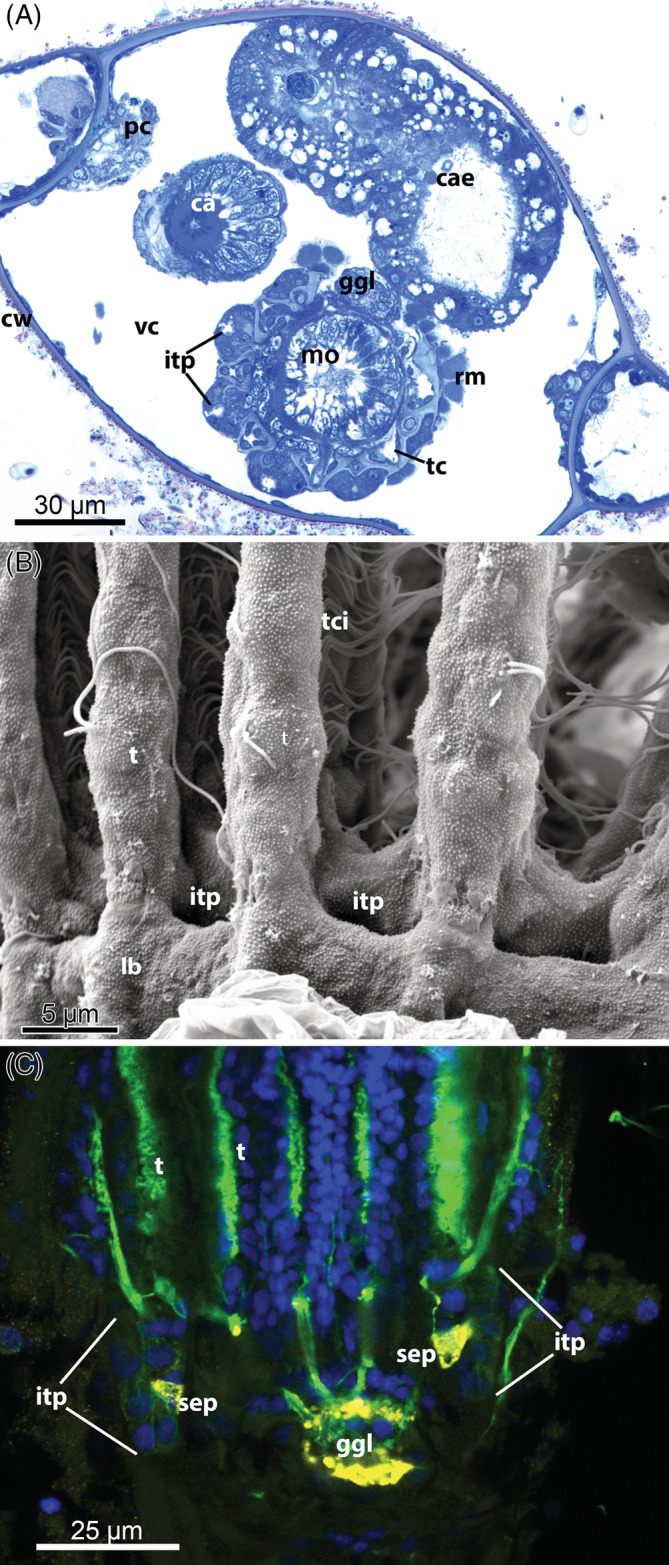
Intertentacular pits of the Gymnolaemata. (A) Lophophoral base of the ctenostome *Victorella pavida*, retracted condition (semithin cross section). (B) Lophophoral base of the ctenostome *Paludicella articulata*; view from the abfrontal side (scanning electron micrograph). (C) Retracted lophophore of the cheilostome *Lanceopora* sp. Confocal laser scanning microscopy, optical section with staining for acetylated alpha‐tubulin (green), serotonin (yellow) and cell nuclei (blue). Abbreviations: ca, cardia; cae, caecum; cw, cystid wall; ggl, ganglion; itp, intertentacular pit; lb, lophophoral base; mo, mouth opening; pc, pore‐cell complex; rm, retractor muscle; sep, serotonin‐like immunoreactive perikaria embedded in the intertentacular pits; t, tentacle; tc, tentacle coelom; tci, tentacle cilia; vc, visceral cavity/coelom.

G6: *Intertentacular pits* (Fig. 10). In the cheilostome *Cryptosula pallasiana* there are pits between the tentacle bases that run into narrow grooves between the tentacles (Gordon, 1974). Cilia have been detected at the bottom of these pits, suggesting a sensory function. In addition, serotonin‐like immunoreactive (serotonin‐lir) perikarya are commonly situated in these pits (Schwaha & Wanninger, 2015; Fig. 10C). Recently these pits have been described in two ctenostome species, *Hislopia malayensis* and *Paludicella articulata* (Schwaha & Wood, 2011; Weber *et al*., 2014), and it now appears that they may be widespread among Ctenostomata and Cheilostomata (T. F. Schwaha, personal observations).

G7: *Intertentacular organ*. A number of ctenostomes from different families as well as membraniporine‐grade ‘basal‐branching’ cheilostomes release their eggs *via* a two‐chambered tube with internal ciliation at the anal side of the tentacle crown (see Temkin, 1994; Reed, 1991). In the vast majority of such forms the eggs develop into non‐brooded planktotrophic larvae, suggesting that this character is a symplesiomorphy of Gymnolaemata. An alternative hypothesis proposes an independent origin of the intertentacular organ in two gymnolaemate lineages (Ostrovsky & Porter, 2011).

G8: *Cyphonautes larva*. There are two distinct larval types in Gymnolaemata: the planktotrophic cyphonautes larva and the lecithotrophic (endotrophic) coronate larva. The cyphonautes larva is distributed among various ctenostome genera as well as in membraniporine ‘basal‐branching’ cheilostomes (Wood, 2008; Nielsen & Worsaae, 2010; Ostrovsky, 2013*a*) and has a relatively uniform structure. Given its patchy distribution, it appears likely that this larval type was ancestral for Gymnolaemata (Zimmer & Woollacott, 1977*a*). The non‐feeding coronate larvae show much higher morphological variability (Zimmer & Woollacott, 1977*b*; Temkin & Zimmer, 2002; Santagata, 2008; Gruhl, 2009). They are always incubated and (together with incubation chambers) are thought to have evolved multiple times from a feeding larval type in Gymnolaemata (Taylor, 1988; Ostrovsky, 2013*a*). A number of intermediate larval forms exist with a dysfunctional gut in some ctenostome and cheilostome species (Zimmer & Woollacott, 1977*b*).

G9: *Reduced peritoneal lining of the body wall*. In a strict sense, the coelomic cavity should be lined by a complete peritoneal layer (epithelium) with the apical sides of the cells facing the fluid‐filled cavity. This condition is found within the Phylactolaemata (see Mukai *et al*., 1997; Gruhl *et al*., 2009) as well as Stenolaemata with their membranous sac (see character S2 in Section II.1*d*). Although a distinct layer of peritoneal palisade cells is present in the body wall of the developing cystid (Tavener‐Smith & Williams, 1972), there are several accounts that describe the peritoneal layer as incomplete, patchy and diffuse, or even missing in gymnolaemates (e.g. Borg, 1926; Lutaud, 1983; Woollacott & Zimmer, 1975; Mukai *et al*., 1997; Shunatova & Tamberg, 2019). This implies that almost all adult gymnolaemates are actually acoelomate. A complete peritoneal lining is present, however, covering the parts of the polypide, and nutrient storage cells above the epidermis of the body wall (Hughes, 1987; Nekliudova *et al*., 2019*a*,*b*; Shunatova & Tamberg, 2019) are probably of peritoneal origin too.

#### (f) *‘Ctenostomata’*

As a paraphyletic assemblage of non‐calcified gymnolaemates (e.g. Fig. 3C), there is no morphological character that can be used to define a ctenostome bryozoan other than the general features given above. The recognition of seven or eight superfamilies is based either on cystid or colony traits (or both) (see Todd, 2000). A molecular study of this small group is still in its infancy (Waeschenbach *et al*., 2012, 2015), and soft‐part morphological studies are also scarce (Jebram, 1973*a*, 1986*a*,*b*; Schwaha & Wanninger, 2018).

#### (g) *Cheilostomata (Branch F in Fig. 2)*

C1: *Operculum* (Figs 3D, 8A–C). A flap‐like outfold of the body wall providing closure of the zooidal orifice after lophophore retraction. It is supported by a strong, chitinous opercular rim that is generally non‐calcified (Taylor & Zagorsek, 2011). A similar structure is present in a few ctenostomes: e.g. the fossil *Cardioarachnidium bantai* (Taylor, 1990) and in recent species of the genus *Penetrantia* (Soule & Soule, 1969, 1975). In some cheilostomes, the operculum has a calcified internal wall (cryptocyst) (Banta, Gray & Gordon, 1997). A calcified hinged operculum evolved independently in an extinct group of cyclostomes (Taylor, 1985).

C2: *Calcified skeleton*. All cheilostomes have a calcified skeleton acquired independently from that of Cyclostomata (see Section II.1*d*).

C3: *Complex funicular system*. The funicular system of cheilostomes consists of a complex intrazooidal network of anastomosing peritoneal/mesodermal strands (with or without internal lacunae) that interconnects zooids within the colony and distributes nutrients between them (Bobin, 1977; Lutaud, 1982; Best & Thorpe, 1985). These strands emanate from the peritoneal lining of the gut and run to pore‐cell complexes of the communication pores in the interzooidal walls or to the lateral funicular strands in the vicinity of the body wall. In some species the funicular tissue contains bacterial symbionts in so‐called ‘funicular bodies’ (Lutaud, 1969; Mathew *et al*., 2018; Shunatova & Tamberg, 2019; Karagodina *et al*., 2018).

C4: *Multiporous septula between zooids* (Fig. 8D). Communication organs in cheilostomes are commonly multiporous, i.e. each lateral or transversal wall is pierced by several groups of pores (termed multiporous septula) that interconnect adjacent zooids (Banta, 1969; Bobin, 1977; Ostrovsky, 1998; Mukai *et al*., 1997). Two ctenostome genera, *Pherusella* and *Sundanella*, also have multiporous septula (Osburn, 1953; Marcus, 1941).

C5: *Vestibular glands* (Fig. 8C). Sac‐like glands are formed as invaginations of the vestibular wall of autozooids and some heterozooids (avicularia) (Waters, 1892; Lutaud, 1964, 1965, 1986). These glandular bodies, which can harbour symbionts, have been recorded in numerous cheilostomes, although their function is not clear (Lutaud, 1965, 1986). Similar glands also have been found in the ctenostome genus *Penetrantia* (Soule & Soule, 1975).

### (2) Trends in structural and functional evolution

The apomorphies described above are consistent with the topology of the major bryozoan clades reconstructed in the latest molecular tree (Waeschenbach *et al*., 2012). Despite uncertain polarity for many characters, we can now discuss the various levels of complexity observed in the different clades in terms of their evolution and variation. In this section, we analyse comparatively the organ systems in Bryozoa in order to reveal major trends in their morpho‐functional evolution. This yields new insights into the morphological characters of the last common bryozoan ancestor and the evolution of these characters within the phylum.

#### (a) *Lophophore retraction and protrusion*

##### (i) *Retraction*

**Figure 11:**
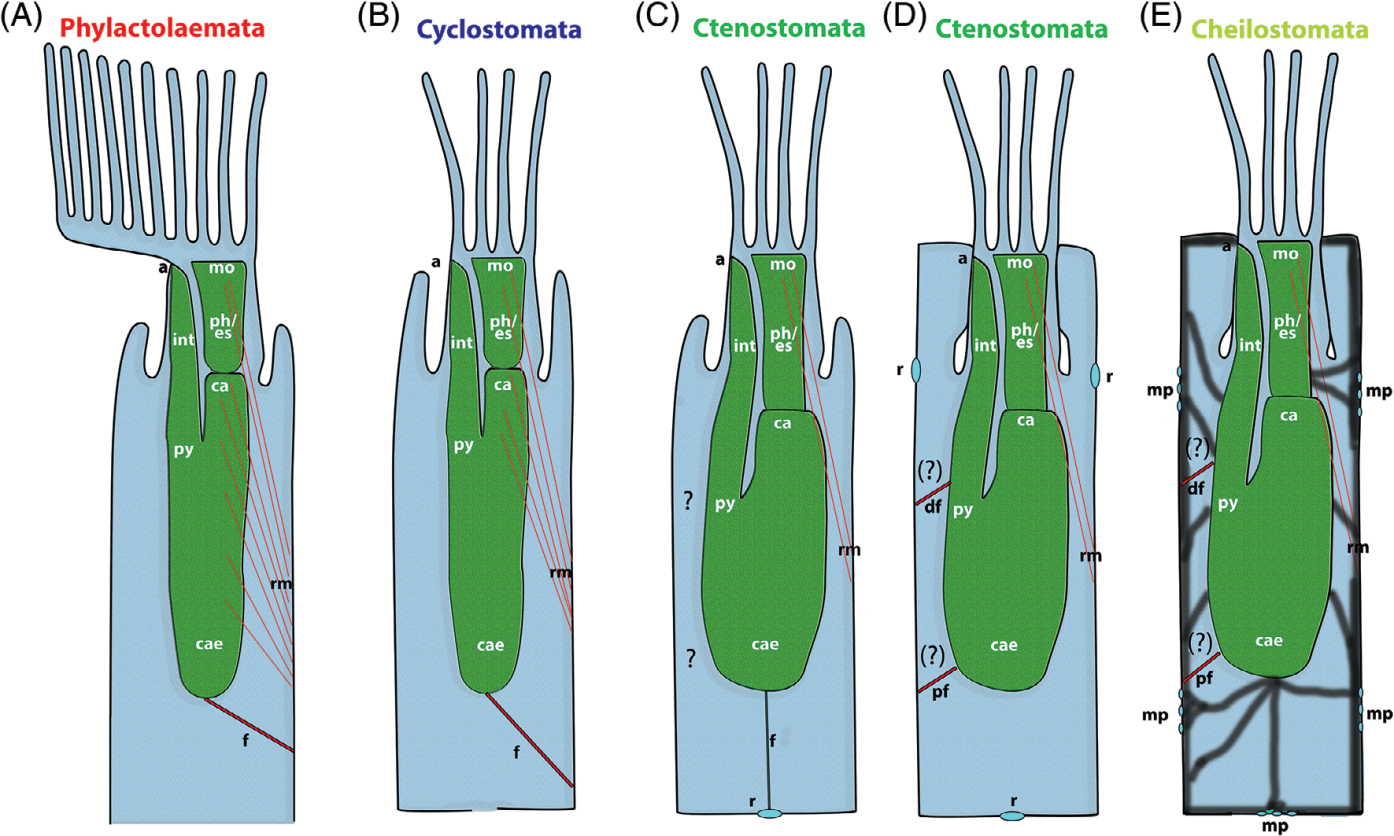
Schematic sections through a single zooid of the different bryozoan taxa showing retractor muscle insertions on the polypide as well as proposed funicular homologues (see main text for details). Parts of the gut and interzooidal communication are also depicted. Proportions of gut and apertural area are shown schematically and not to scale. (A) Phylactolaemata characterized by a horseshoe‐shaped lophophore show retractor muscle fibres attached over the entire foregut and a simple funiculus (peritoneal tubular strand with longitudinal muscles). (B) Cyclostomata show a reduction in the number of retractor muscles which only insert up to the cardia and a simple funiculus. (C) In the Gymnolaemata retractors are attached exclusively to the lophophoral base. Ctenostomes with polymorphic stolons, which are commonly depicted as ‘generalized’ Ctenostomata in most textbooks, are derived forms (Jebram, 1973*a*; Todd, 2000). They possess a funiculus as a communication organ interconnecting zooids *via* pore‐plates within the colonies. The condition of possible caecal ligaments/funicular strands directed towards the body wall remains questionable. (D) Ctenostomes without stolons and with flat, encrusting colonies show individual zooids with four rosette pore plates not connected by the funicular strands. Current data indicate that the muscular function of either of the two cords may be lost or the cords may be reduced (question marks). (E) Membraniporine cheilostome with multiporous septula and funicular cords similar to those found in ctenostomes (probably representing the original funicular cords). The complex anastomozing network of mesodermal strands is considered a new feature that enhanced and facilitated interzooidal/intracolonial communication and integration. Abbreviations: a, anus; ca, cardia; cae, caecum; df, distal funiculus; f, funiculus; int, intestine; mo, mouth opening; mp, multiporous pore plates; pf, proximal funiculus; ph/es, pharynx‐oesophagus; py, pylorus; r, rosette pore plate; rm, retractor muscle.

The movement of the polypide into and out of the cystid is a common feature of Bryozoa. Retraction is caused by the largest muscles in the zooid – paired retractors, placed on each side of the digestive tract (Fig. 11). In the Phylactolaemata each retractor muscle consists of several bundles that insert at: (*i*) the lophophoral base/parts of the tentacle sheath; (*ii*) the pharynx, oesophagus or cardia of the gut; and (*iii*) various parts of the caecum (Allman, 1856; Hyatt, 1865; Kraepelin, 1887). As a result, retraction drags not only the lophophore, but the whole polypide over its entire length into the cystid.

In other bryozoan clades, there appears to be a stepwise reduction in the number of retractor muscle bundles. In the Cyclostomata, in addition to the bundles projecting to the lophophoral base, some species show a short single bundle running towards the oesophagus‐cardia area (Nielsen & Pedersen, 1979; Schäfer, 1985; Boardman, 1998). In the Gymnolaemata, the retractor muscle fibres generally only attach at the lophophoral base (although exceptions have been described recently; Schwaha *et al*., 2019b). These structural differences ultimately lead to a different arrangement of the gut in a retracted polypide. In the Phylactolaemata, the mouth and anus are located at about the same level. At least in Gymnolaemata, the anus always lies more distally than the mouth opening after polypide retraction.

##### (ii) *Protrusion*

**Figure 12:**
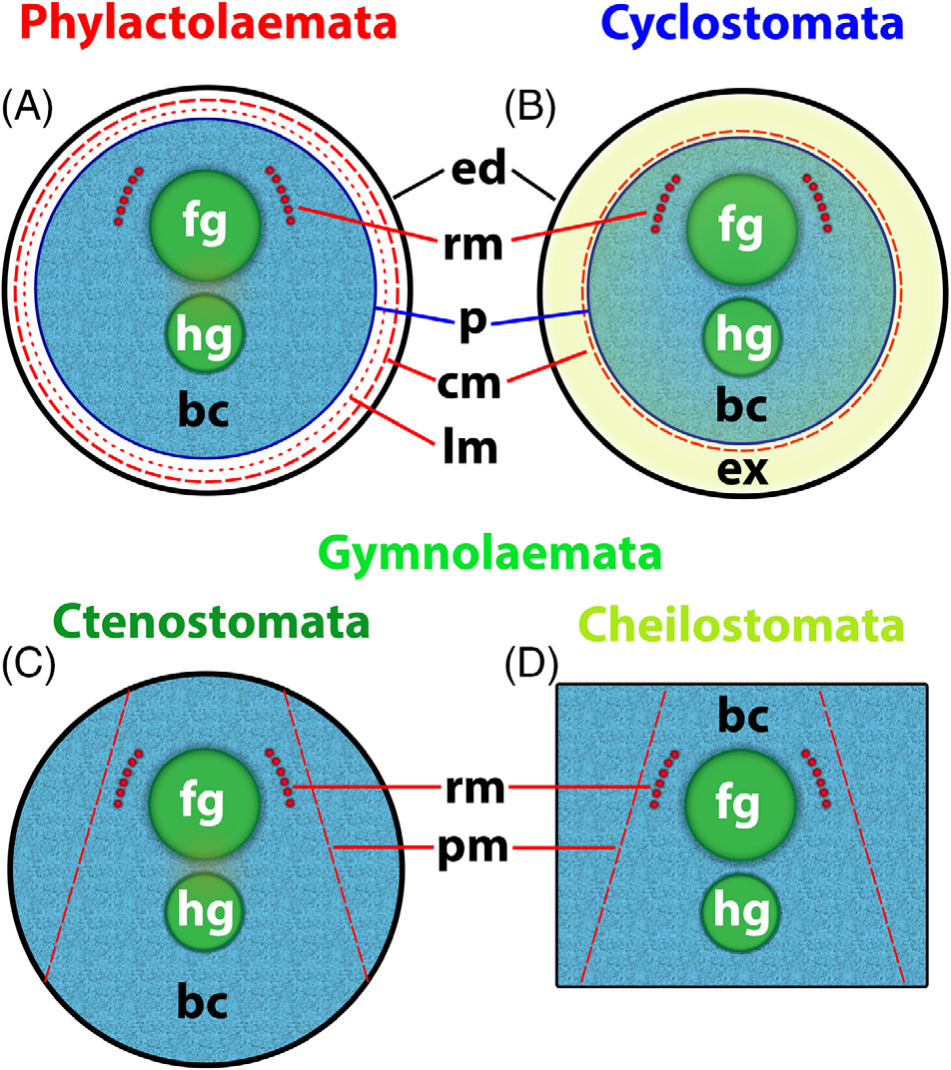
Schematic cross sections of single autozooids to show the condition of the body wall and associated musculature in the different bryozoan taxa [sections show ascending and descending part of the gut; modified and redrawn from Jebram (1986*a*)]. (A) The Phylactolaemata possess both circular and longitudinal musculature between the epidermis and the peritoneum of the body wall. (B) In the Cyclostomata the peritoneum has detached from the epidermal layer, creating the exosaccal space in between. The peritoneum forms the membranous sac around the polypide and is supplied by annular ring muscles derived from the original body wall musculature. (C, D) In the Gymnolaemata the peritoneal layer in the body wall probably has been reduced to an incomplete epithelium. Parts of the original circular musculature of the body wall now traverse the body cavity in the form of parietal muscles. (D) Cheilostomes commonly have a more box‐shaped cystid. A similar morphology is also present among several encrusting ctenostomes. Abbreviations: bc, body cavity; cm, circular musculature; ed, epidermis; ex, exosaccal space; fg, foregut; hg, hindgut; lm, longitudinal musculature; p, peritoneum; pm, parietal musculature; rm, retractor muscle.

Unlike the retraction process, different mechanisms are employed by the different clades for polypide protrusion [see also summaries in Taylor (1981) and Mukai *et al*. (1997)]. Phylactolaemates have regular body wall musculature (Figs 11 and 12) which contracts the flexible cystid wall leading to an increase in hydrostatic pressure within the coelomic cavity that pushes out the retracted polypide.

Cyclostomes have a membranous sac with annular ring muscles whose contraction results in an increase in coelomic fluid pressure within the internal sac that pushes out the polypide (Nielsen & Pedersen, 1979; Taylor, 1981; Mukai *et al*., 1997; Schwaha *et al*., 2018). These muscles are probably derived from the circular musculature of the original body wall musculature (Nielsen & Pedersen, 1979) although Taylor (1981) suggested that they originated from parietal muscles of a pro‐ctenostome ancestor.

Gymnolaemates possess paired transversal parietal muscles that traverse from the lateral or basal walls to the frontal wall. There are major modifications in the number and position of these muscles in the Cheilostomata depending on frontal wall calcification and the structure of the compensatory sac when present (Cheetham & Cook, 1983; Gordon & Voigt, 1996; Banta *et al*., 1997; Mukai *et al*., 1997). The principal protrusion mechanism in the Gymnolaemata remains the same, i.e. compressing uncalcified parts of the cystid to increase the hydrostatic pressure within the coelomic/visceral cavity to push out the polypide. As previously noted, the parietal muscles are likely to be derived from the ancestral circular musculature (‘displaced body‐wall musculature’ in Hyman, 1959), and in this context represent an economic and effective mechanism for polypide protrusion (Jebram, 1986*a*).

In summary, two evolutionary trends are recognizable when comparing muscular systems among different bryozoan clades. In comparison to Phylactolaemata, the number of retractor muscle bundles was reduced in the Myolaemata, and the circular muscular layer of the body wall was independently reduced to annular muscles in Cyclostomata and transversal parietal muscles in Gymnolaemata.

#### (b) *Digestive tract*

**Figure 13:**
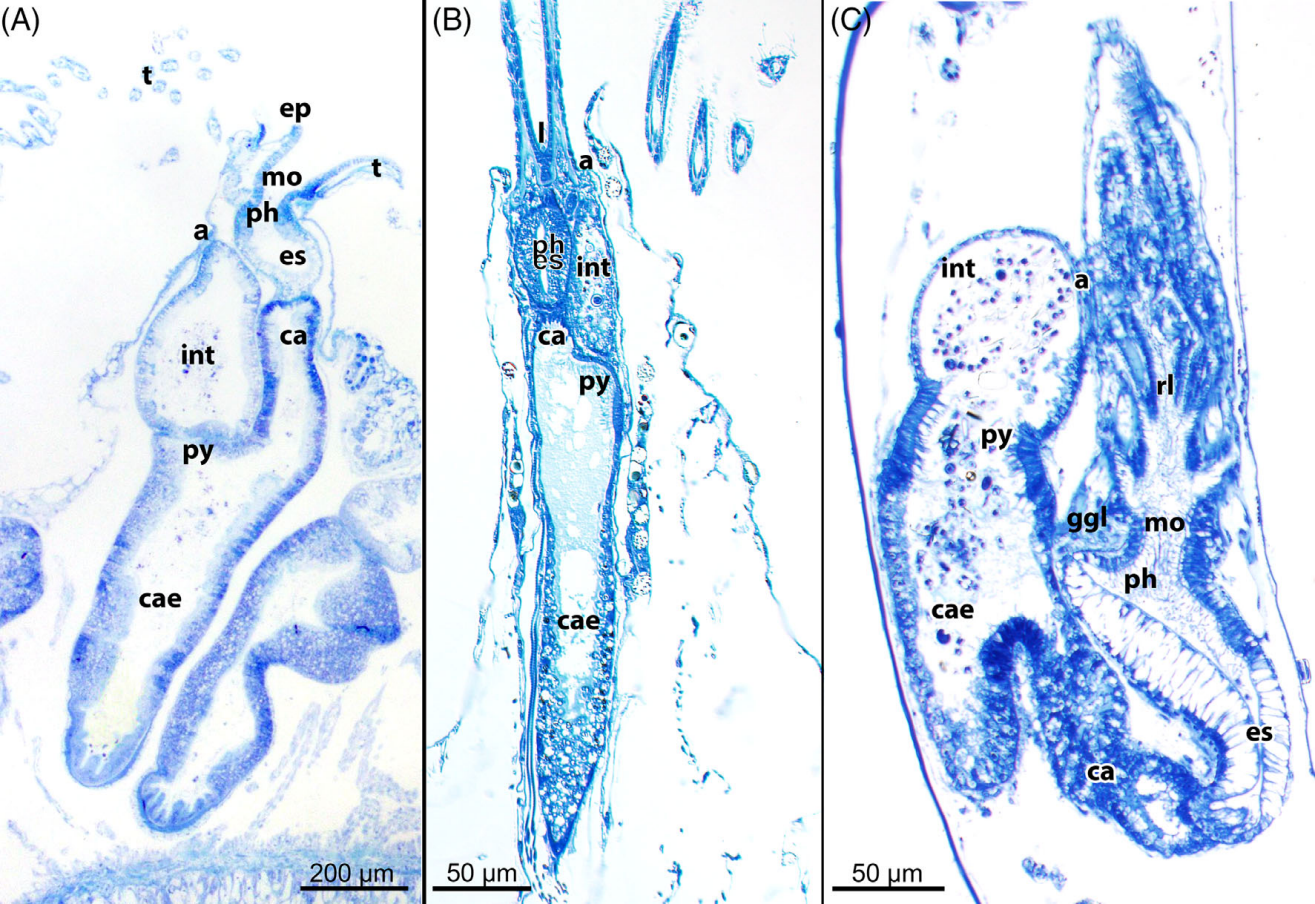
Digestive tract in Bryozoa showing the different parts of the foregut (mouth opening, pharynx, oesophagus), midgut (cardia, caecum, pylorus) and hindgut (intestine, anus) (semithin longitudinal sections). (A) Phylactolaemate condition exemplified by *Cristatella mucedo*. (B) Cyclostome condition exemplified by *Patinella radiata*. (C) Gymnolaemate condition exemplified by the cheilostome *Bugula neritina*. The foregut (pharynx and oesophagus) as well as the cardia are commonly elongated in Gymnolaemata. Abbreviations: a, anus; ca, cardia; cae, caecum; ep, epistome; es, oesophagus; ggl, ganglion; int, intestine; l, lophophore; mo, mouth opening; ph, pharynx; py, pylorus; rl, retracted lophophore; t, tentacle.

Although the first descriptions of the bryozoan digestive tract date from the 18th century (e.g. Trembley, 1744), there have been few detailed comparative studies of its structure (e.g. van Beneden, 1845*a*,*b*; Smitt, 1865; Hyatt, 1865; Calvet, 1900; Borg, 1926; Marcus, 1934; Braem, 1940*a*, 1951; Silén, 1944*a*; Gordon, 1975*b*; Mukai *et al*., 1997). The mouth opening at the lophophoral base leads into the ciliated pharynx, which in different clades shows structural and functional variation (e.g. myoepithelial suction pump and triradiate lumen in Myolaemata). The pharynx is followed by a non‐ciliated oesophagus. The oesophagus adjoins the cardia which represents the first part of the stomach. To prevent reflux of nutrients during the retraction process, a cardiac valve is present at the oesophagus–cardia border (see Gordon, 1975*b*; Schwaha & Wood, 2011; Schwaha, 2019*b*). The cardia enters the voluminous caecum where most digestion takes place, followed by a short pylorus and an intestine which terminates in the anus outside the lophophoral crown (Figs 11 and 13). The presence of the cardiac valve is reflected in polypide ontogeny: early in the two‐layered bud, two invaginations, the prospective mouth area and the prospective anal area, grow towards each other. From the prospective mouth area the foregut (pharynx and oesophagus) develops whereas all the remaining parts of the digestive tract differentiate from the prospective anal area (see Braem, 1890; Borg, 1926; Lutaud, 1983; Schwaha *et al*., 2011a; Schwaha & Wood, 2011). The boundary where these two anlagen meet and fuse always represents the cardiac valve.

In non‐gymnolaemates the oesophagus and cardia are usually rather short, but in the Gymnolaemata these parts usually form an elongated tube (Borg, 1926; Silén, 1944*a*). The contribution from the prospective anal area (cardiac portion) and from the prospective mouth area (oesophageal portion) can vary among different species – although only a few species have been analysed in detail (Schwaha & Wood, 2011). The position of the anti‐reflux cardiac valve might have functional implications, perhaps correlated with digestion time, but these are currently unknown.

A putative apomorphy of the Myolaemata is that the pylorus is ciliated and acts in food transport as well as in faecal pellet formation in the hindgut (Silén, 1944*a*; Winston, 1977). This is in strong contrast to phylactolaemates in which the pyloric area is unciliated and which have a different mode of internal food manipulation: food particles are kneaded up and down the caecum by a dense array of striated muscle fibres (Mukai *et al*., 1997; Schwaha & Wanninger, 2012). In addition, the digestive epithelium is folded into several ridges giving the caecal lumen a star‐shaped appearance in cross section in Phylactolaemata (e.g. Borg, 1926; Mukai *et al*., 1997). This increases the surface area of the digestive epithelium and facilitates tighter contact with food particles. Gymnolaemates and cyclostomes both lack these ridges, and food particles are not in direct contact with the epithelium. Instead, the rotary action of the ciliated pylorus creates a rotating mass of food within the stomach where digestion takes place (Silén, 1944*a*; Winston, 1977). The muscular net surrounding the stomach is much sparser in these clades than in Phylactolaemata and mainly consists of only a few smooth fibres (Gordon, 1975*b*; Mukai *et al*., 1997; Schwaha *et al*., 2011b, T. F. Schwaha, personal observations), which mainly act in moving particles from the caecum towards the cardia or pylorus (Silén, 1944*a*).

In addition, in some cyclostomes and gymnolaemates (both ctenostomes and cheilostomes), part of the cardia can be modified to form a gizzard with chitinous denticles or a cuticular proventriculus (Braem, 1951; Gordon, 1975*a*; Schäfer, 1986; Markahm & Ryland, 1987). It was suggested that the gizzard is an ancestral ctenostome character and was subsequently reduced multiple times (Jebram, 1973*a*). However, in the absence of a proper phylogeny for this taxon as well as additional data on gut anatomy in different ctenostome clades, it is currently impossible to assess whether the gizzard is an ancestral ctenostome and thus also a gymnolaemate character. In summary, the digestive tract of the Myolaemata is quite different from the phylactolaemate condition, resulting in a different mode of food manipulation. As with many characters, however, neither can be currently designated as an ancestral type.

#### (c) *Nervous system*

The nervous system of bryozoans is subepithelial and has its centre in the cerebral ganglion or brain located at the anal side of the pharynx. The ganglion contains a lumen in all phylactolaemates and a few myolaemates, and a circum‐oral nerve ring emanates from both lateral sides of the ganglion to pass around the pharyngeal wall/lophophoral base towards the oral side. In most studied species it forms a closed ring on the oral side. The innervation of the lophophore including the tentacles comes from the ganglion and the circum‐oral nerve ring, whereas the remaining two larger neuronal systems, one entering the tentacle sheath and the second entering the digestive (visceral) tract, emerge directly solely from the cerebral ganglion. The tentacle sheath neurite bundles extend over the apertural area towards the body wall (Schwaha, 2019*b*).

A comparative analysis of the available data revealed some trends in the evolution of the bryozoan nervous system that concern the tentacle neurite bundles and their origins as well as the neurite bundles of the tentacle sheath and the visceral innervation. Recently, new data emerged on an outer ring neurite bundle that occurs in phoronids, brachiopods and few selected bryozoans (Temereva, 2017*a*,*b*).

(1) Tentacle neurite bundles in the Bryozoa are located frontally, i.e. facing the mouth opening, and abfrontally, i.e. facing the side opposite to the mouth opening. In the Phylactolaemata most recent studies have identified six tentacle neurite bundles – three frontal (one medio‐frontal and two latero‐frontal) and three abfrontal (one medio‐abfrontal and two latero‐abfrontal) (Shunkina *et al*., 2015; Ambros, Wanninger & Schwaha, 2018). In other studies, three abfrontal, two latero‐frontal and 2–5 medio‐frontal neurite bundles have been reported (Mukai *et al*., 1997; Tamberg & Shunatova, 2017). We consider the latter as bundles representing one (although complex) frontal neurite bundle. Cyclostomes and gymnolaemaetes almost invariably possess three distinct neurite bundles on the frontal side, suggesting that the three frontal ones (one medio‐frontal and two latero‐frontal) represent the ground pattern of Bryozoa. In the cyclostome *Cinctipora elegans*, the mediofrontal bundle terminates soon after its emergence or joins the latero‐frontal ones (Schwaha *et al*., 2018), whereas it is continuous in the cyclostome *Crisia eburnea* (Temereva & Kosevich, 2018). Besides the three frontal neurite bundles, three abfrontal ones were detected in *Crisia eburnea* (Temereva & Kosevich, 2018) whereas only a single abfrontal nerve was detected in all gymnolaemates and the cyclostome *Cinctipora elegans* (Smith, 1973; Lutaud, 1973; Gordon, 1974; Nielsen & Riisgård, 1998; Schwaha & Wood, 2011; Weber *et al*., 2014; Schwaha *et al*., 2018). Since the latter was not studied using transmission electron microscopy, small latero‐abfrontal neurite bundles might have been overlooked.

Recently, only two tentacle neurite bundles (frontal and abfrontal) were reported in the ctenostome *Amathia gracilis* (Temereva & Kosevich, 2016). The frontal neurite bundle, however, is a result of the fusion of the two latero‐frontal and the medio‐frontal neurite bundles at the tentacle base. Since latero‐abfrontal neurite bundles were found in all phylactolaemates and a cyclostome, it can be concluded that these were likely in the ground pattern of bryozoans and have been lost in gymnolaemates and probably some cyclostomes. However, further data are required to corroborate the general presence of these bundles in cyclostomes.

In general, the distribution of the three frontal neurite bundles correlates with the three rows of frontal ciliary cells, i.e. medio‐frontal and two latero‐frontal, as well as the stiff, probably sensory cilia on the abfrontal side (see Schwaha, 2019*b*). The specific role of the latero‐abfrontal neurite bundles and their association with any ciliary structures has not been analysed.

(2) The tentacle neurite bundles in the Phylactolaemata branch off the so‐called radial nerves intertentacularly and further emanate into the tentacles (Gerwerzhagen, 1913*a*; Lutaud, 1977; Shunkina *et al*., 2015; Ambros *et al*., 2018). In recently studied ctenostomes all but one (the medio‐frontal neurite bundles projecting from the circum‐oral nerve ring) have an intertentacular origin from radial nerves (Schwaha & Wood, 2011; Weber *et al*., 2014; Temereva & Kosevich, 2016; Schwaha, 2019*b*). In the Cheilostomata only the two latero‐frontal neurite bundles have an intertentacular origin, whereas the two others are described to emanate from the circum‐oral nerve ring (Lutaud, 1973, 1977). However, the latter have not been studied using modern techniques. Preliminary observations show that distinct asymmetries are present in the tentacle nerve branching that give the impression of direct neurite bundles, but in fact are single roots emerging intertentacularly (T. F. Schwaha, personal observations).

Few studies have been conducted on the tentacular innervation pattern of Cyclostomata (Schwaha *et al*., 2018; Temereva & Kosevich, 2018; Worsaae, Frykman & Nielsen, 2019). The available data show that the ramification pattern of tentacle neurite bundles in cyclostomes is very similar to the gymnolaemate condition. The latero‐frontal neurite bundles have an intertentacular origin whereas the medio‐frontal neurite bundles either emanate directly from the circum‐oral nerve ring in the median axis of the tentacle or have two short rootlets from an intertentacular position before fusing in the median tentacle plane (Schwaha *et al*., 2018; Temereva & Kosevich, 2018; Worsaae *et al*., 2019). The abfrontal neurite bundles show some variation in their origin, but there is a general tendency in all bryozoans of asymmetries and variations in these bundles (Ambros *et al*., 2018; T. F. Schwaha, unpublished data).

It is evident that there is a full complement of intertentacular neurite origins in Phylactolaemata with a trend towards a more direct origin of neurite bundles from the circum‐oral nerve ring in Myolaemata. This probably reflects the smaller size of the myolaemate lophophore including its base. The distance from the circum‐oral nerve ring to the lophophoral base is short in myolaemates and much longer in phylactolaemates. The shorter distance in myolaemates is probably reflected by the direct neurite bundle origin. The high degree of variability and asymmetries in these ramifications remain poorly understood.

(3) Both the tentacle sheath nerves and the visceral (i.e. foregut) innervation show a rather diffuse plexus in Phylactolaemata (Gerwerzhagen, 1913*a*; Shunkina *et al*., 2015; Ambros *et al*., 2018). Within this plexus, the longitudinal neurite bundles are commonly thicker and less numerous, whereas interconnecting transversal and diagonal bundles are shorter and thinner. Gymnolaemates and cyclostomes generally show condensed and regular patterns of the tentacle sheath and foregut innervation rather than a diffuse plexus (Lutaud, 1977; Mukai *et al*., 1997; Weber *et al*., 2014; Temereva & Kosevich, 2016, 2018; Schwaha *et al*., 2018).

(4) An ‘outer ring nerve’ was first recognized in the ctenostome *Amathia gracilis*, comprising a second ring of neurite bundles emanating from the cerebral ganglion (Temereva & Kosevich, 2016). This neurite bundle traverses to a similar extent as the circum‐oral nerve ring, but is situated more proximally, on the outer side of the lophophore base. This outer ring nerve is also present in other ctenostomes (Schwaha, 2019*b*) and the cyclostome *Crisia eburnea* (Temereva & Kosevich, 2018). A topologically identical, albeit mostly incomplete nerve ring is also present in the cyclostome *Cinctipora elegans*, and similar roots emerging from the cerebral ganglion are present in other gymnolaemates in the form of the so‐called ‘trifid nerve’ (Schwaha *et al*., 2018; Schwaha, 2019*b*). This implies that this second outer nerve ring was present in the myolaemate ancestor and has been partially reduced to an incomplete ring (in some cyclostomes and ctenostomes) or reduced to its roots that remain evident as the trifid nerve in all gymnolaemates (see Schwaha *et al*., 2018). Due to the similar innervation of the lophophoral base by two nerve rings in phoronids and brachiopods, it has been argued that the outer nerve ring was present in the last common ancestor of bryozoans (Temereva & Kosevich, 2016). This would imply that phylactolaemaetes have lost this neuronal character.

#### (d) *Funicular system*

##### (i) *The common funiculus in major bryozoan clades*

**Figure 14:**
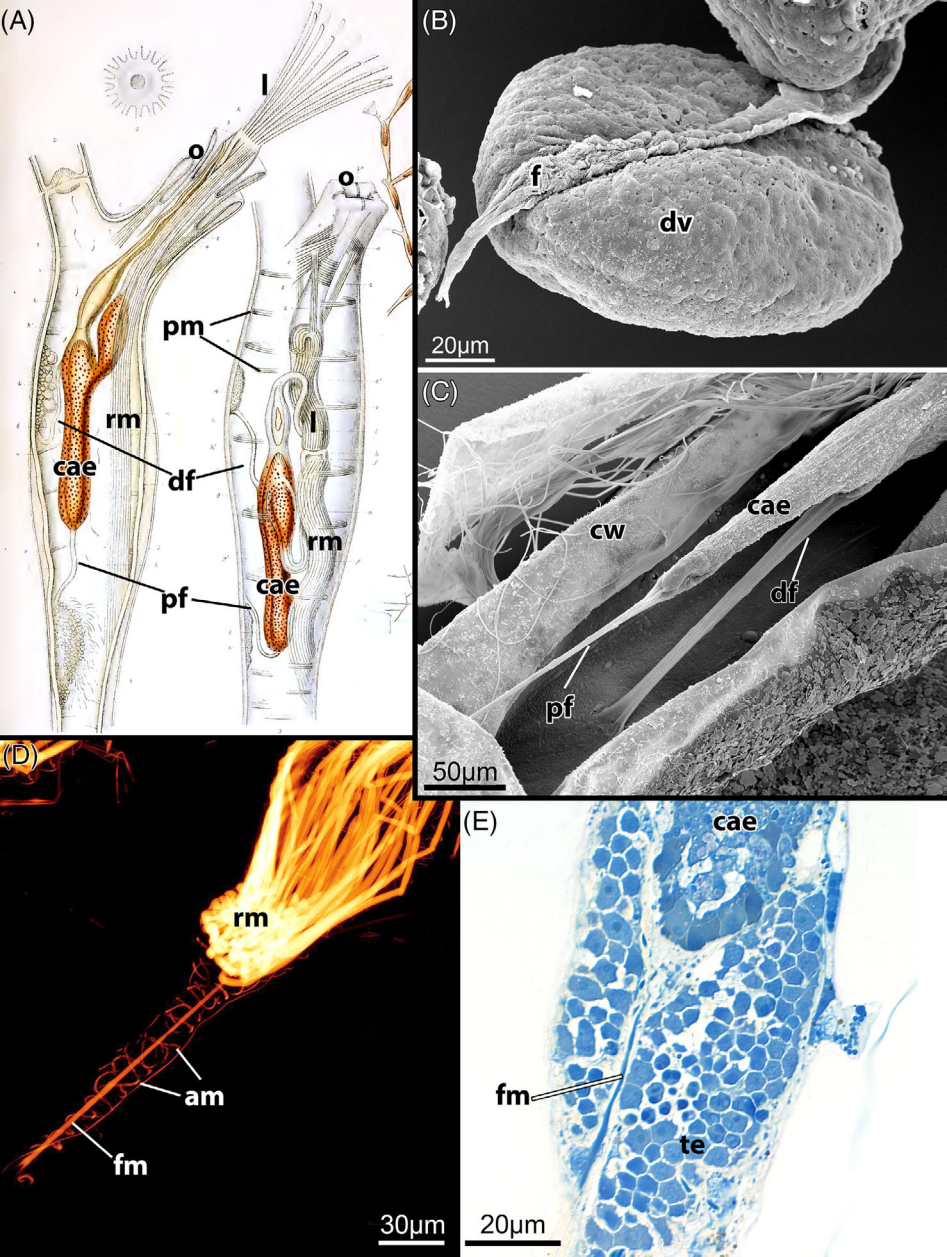
Funicular system in Bryozoa. (A) Schematic drawing of a protruded and retracted polypide of the ctenostome *Paludicella articulata* showing two funicular cords, one proximal and one distal (from Allman, 1856). (B) Scanning electron micrograph (SEM) of the funicular cord of the phylactolaemate *Hyalinella punctata* with developing statoblast inside. (C) SEM of two funiculi of the ctenostome bryozoan *P. articulata*. (D) Muscular funiculus of the cyclostome *Tubulipora* sp. Note also the annular circular muscles of the membranous sac. Optical section, confocal laser scanning micrograph with staining for f‐Actin. (E) Muscular funiculus in the cyclostome *Crisia* sp. (longitudinal semithin section). Abbreviations: Am, annular muscles; cae, caecum; cw, cystid wall; df, distal funiculus; dv, developing statoblast; f, funiculus; fm, funicular muscle; l, lophophore; o, orifice; pf, proximal funiculus; pm, parietal musculature; rm, retractor muscles; te, testis.

The bryozoan funiculus is a tubular peritoneal cord which is often associated with the gonad(s) (Reed, 1991). In the Phylactolaemata, the funiculus contains a central lumen and also carries the developing floatoblasts and thus can reach an extensive length (Wood, 1983, 2014; Figs 6C and 14B). It is supplied with longitudinal muscles and runs from the proximal tip of the caecum towards the body wall (Carle & Ruppert, 1983; Mukai *et al*., 1997; Schwaha & Wanninger, 2012). Similarly, the cyclostome funiculus is a tubular peritoneal cord with longitudinal musculature that extends from the proximal end of the caecum towards the lateral cystid wall (Nielsen & Pedersen, 1979; Schwaha *et al*., 2018; Fig. 14D, E). In the Gymnolaemata, typical textbook representations show ‘stoloniferan’ ctenostomes, i.e. those possessing long kenozooidal stolons devoid of polypides, with a proximal cord from the caecum towards the communication pore between autozooid and stolon (e.g. Ryland, 1970; Reed, 1991; Mukai *et al*., 1997). In this case the funicular strand connects to a stolonal strand that runs along the stolon towards the communication pores of neighbouring zooids. The cheilostome funicular system is commonly represented by a complex network of peritoneal strands with internal lacunae or channels (e.g. Lutaud, 1962, 1982, 1983; Carle & Ruppert, 1983; Mukai *et al*., 1997). These branching and anastomosing strands run from the peritoneum of the gut towards communication pores in the zooidal walls and thus allow for connection to, and communication with, the corresponding funicular cords of neighbouring zooids *via* pore‐cell complexes. Such interzooidal contacts allow transport of metabolites between colony members, rendering the colony an integrated physiological system (Lutaud, 1985; Best & Thorpe, 1985). This cheilostome funicular network lacks muscles, whereas muscular funicular cords connect the gut with the body wall (Schwaha, 2019*b*; see Section II.2*d*.*iii*).

##### (ii) *Funicular diversity in ctenostomes*

For the funicular system among ctenostomes, an extensive analysis of the literature and our own data allows us to identify several structural variants (see online Supporting Information, Table S1 and Appendix S1). These variants can be summarized as follows: most ctenostome superfamilies show a proximal (posterior) funiculus attached to the caecum and a distal (anterior) funiculus in the area of the pylorus (Figs 11C, D, and 14A, C). Commonly, these are supplied with longitudinal muscles similar to the above described funiculus of phylactolaemates and cyclostomes. Variations exist with sometimes only a single funiculus (proximal or distal) or loss of muscles in the different groups (Table S1). A distinct interconnection of zooids *via* funicular strands passing through the communication pore is, however, not present in most superfamilies but appears restricted to the stoloniferan vesicularioideans, victorelloideans and the family Nolellidae in Arachnidioidea. These are all ctenostomes with very large peristomes that have been reported to possess funicular cords in association with communication pores that act as a colonial system of integration (CSI).

**Figure 15:**
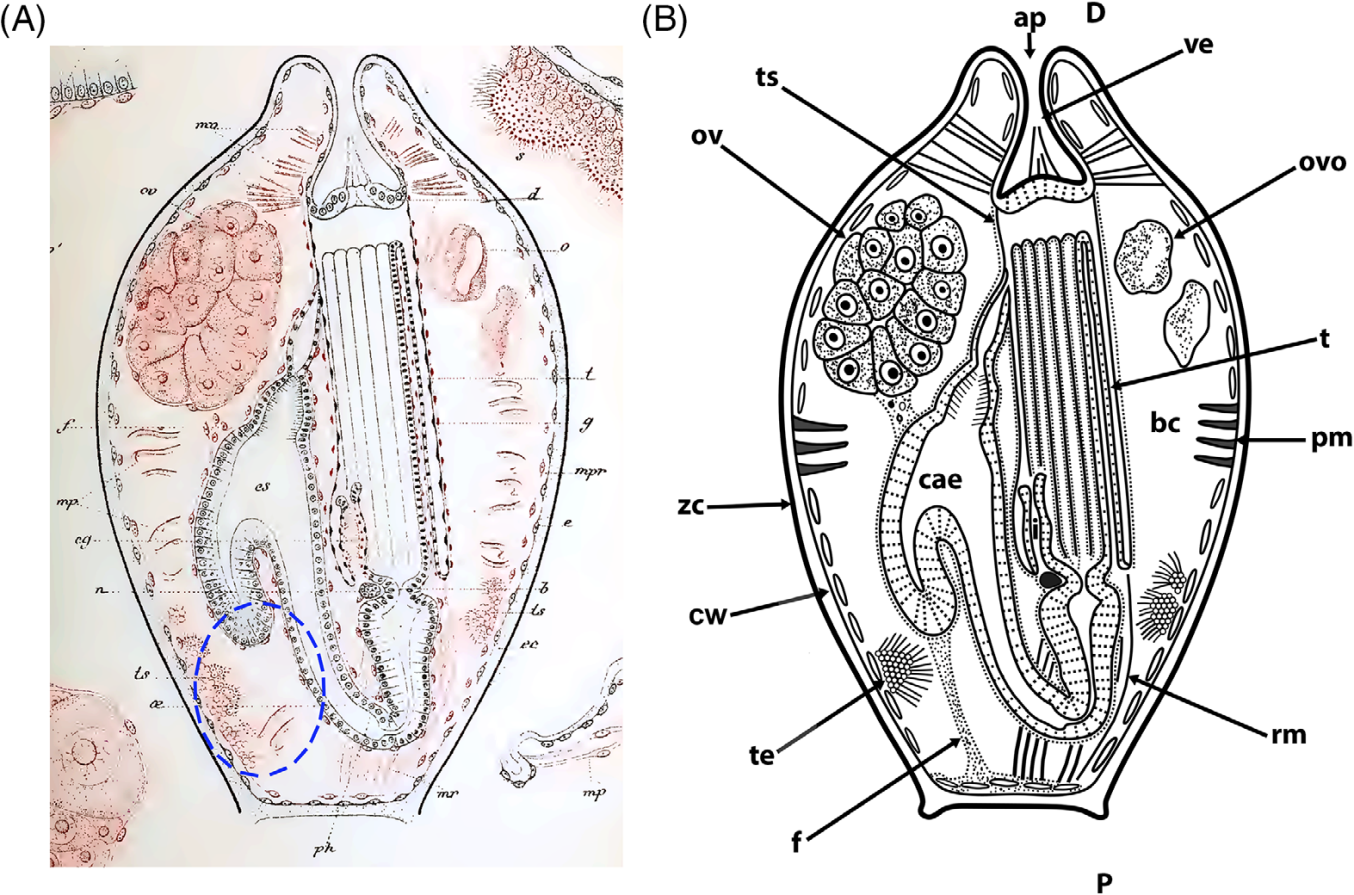
Comparison of a schematic drawing of the ctenostome *Alcyonidium albidum* from (A) the monograph of Prouho (1892) with (B) the redrawn version of Reed (1991). Both drawings show a single retracted zooid with its main components. Note the addition of a proximal funiculus on the right in B that was not present in the original drawing (circled blue line in A). Abbreviations: ap, aperture; bc, body cavity; cae, caecum; cw, cystid wall; D, distal; f, funiculus; i, intertentacular organ; ov, (developing oocytes in) ovary; ovo, ovulated oocytes in the body cavity; P, proximal; pm, parietal muscles; rm, retractor muscles; t, tentacles; te, testis; ts, tentacle sheath; ve, vestibule; zc, zooecium.

Typical textbook examples based on the ‘stoloniferan’ genera *Bowerbankia/Amathia* are valid for the Vesicularioidea, which have large colonies with kenozooidal stolons and attached autozooids. However, our analysis shows much higher diversity and that the general assumption of a funicular CSI is incorrect. The original morphological depiction of *Alcyonidium albidum* (Alcyonidioidea) by Prouho (1892) was altered and redrawn in a more recent compendium with a proximal funiculus running to the communication pore (Reed, 1991; Fig. 15), despite the absence of evidence for such a connection in this species. There appears to be an unsubstantiated assumption that an interconnection between zooids by funicular cords is a common feature to all gymnolaemates. Because four of the eight ctenostome superfamilies lack such funicular communication between zooids, and three of these (Alcyonidioidea, Hislopioidea and Paludicelloidea) are considered to be early‐branching groups in both morphological and molecular analyses (Todd, 2000; Waeschenbach *et al*., 2015), it is more parsimonious to suggest that intracolonial communication *via* funicular strands has evolved independently in some ctenostomes and cheilostomes.

With a simple peritoneal cord with longitudinal muscles, early‐branching ctenostomes have a similar funiculus to that of phylactolaemates and cyclostomes, which can thus be considered plesiomorphic for Gymnolaemata. The two caecal muscular funiculi present in some ctenostomes may represent the result of duplication of the original proximal funiculus. Two such funicular cords attached to the cystid wall are also present in the early‐branching membraniporine cheilostome *Electra* (Fig. 8A, B), whereas most cheilostomes possess only a posterior muscular funiculus (also called caecal ligament) tubular in structure (Lutaud, 1962, 1983). Duplication of the original funiculus may thus have occurred either in the last common ancestor of gymnolaemates (followed by reduction of the distal cord in some ctenostomes and cheilostomes) or independently in these two clades.

##### (iii) *Functional and evolutionary aspects*

Based on developmental, structural and positional criteria it was suggested that the funicular cords of Bryozoa are homologous to the blood vessels of other lophophorates (Carle & Ruppert, 1983). Since the funiculus is associated with gonads that either develop on the cord or lie on the cystid wall, an intrazooidal transport function (which has yet to be proven) from the stomach to the gonads is likely [see e.g. Lutaud (1985)]. However, in sterile zooids of species without interzooidal funicular connections, the funicular cord(s) is/are attached to the cystid wall, and a transport function is not obvious. In the cyclostome *Crisia elongata*, the funiculus is connected by peritoneal (termed ‘mesenterial’) cells to the cells of the neighbouring interzooidal pore in addition to their attachment to the cystid wall (Carle & Ruppert, 1983). This single observation requires verification.

Periodic contractions of the proximal funiculus/caecal ligament were observed in the cheilostome *Membranipora membranacea*, suggesting their participation in food propulsion in addition to peristaltic movements of the gut (Lutaud, 1962). Thus, in this case, the funicular cord may be a contractile organ assisting movements of the digestive tract.

The development of interzooidal funicular connections may have allowed the funiculus to function in transport. From a simple attachment to the cystid wall, the next evolutionary step may have been attachment of the funiculus near the communication pore connecting the cavities of neighbouring zooids or connecting the funiculus to the pore‐cell complex. Although only indirect morphological evidence is present, this appears to be the case in several species of Walkerioidea (Table S1; van Beneden, 1845*b*; Ehlers, 1876; Marcus, 1926*a*). Evidence for transport of nutrients to neighbouring zooids *via* the funiculus is absent in Walkerioidea, and the communication pore itself might serve this purpose (Mukai *et al*., 1997). Such transport could involve the movement of coelomic fluid between zooidal cavities *via* the pore plates.

**Figure 16:**
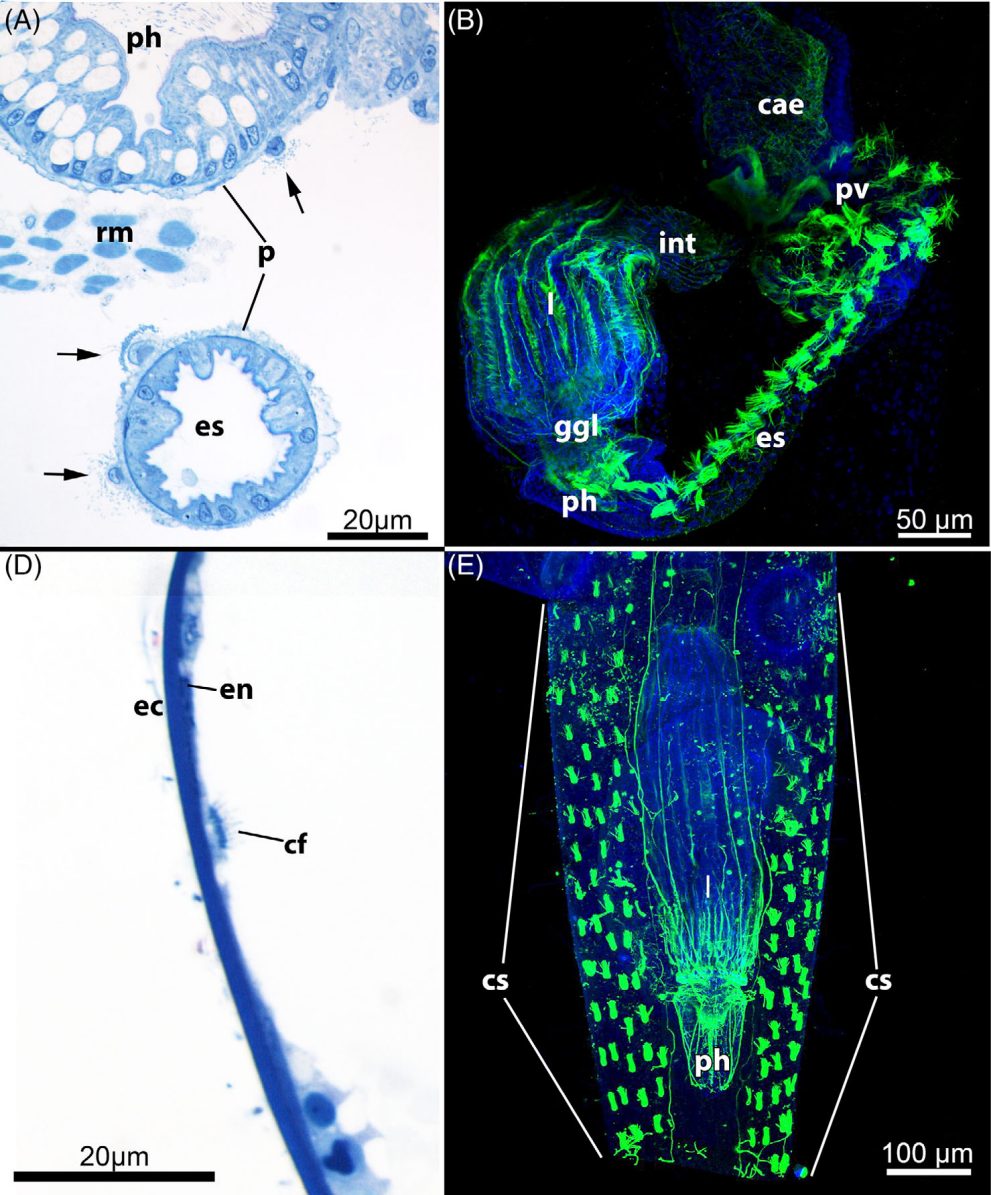
Ciliary structures within the body cavity of two ctenostome gymnolaemates: (A, B) *Hislopia malayensis*; (C, D) *Paludicella articulata*. (A) Foregut, pharynx and oesophagus with ciliary cups on the peritoneal layer (arrows) (semithin cross‐section). (B) Dissected zooid showing arrangement of the ciliary cups on the foregut. Maximum projection of a confocal laser scanning micrograph (CLSM) stack with staining for anti‐acetylated alpha‐tubulin (green) and nuclei (blue). (C) Cystid wall showing a ciliary field of one of the ciliary rows (semithin cross‐section). (D) Lateral arrangement of the ciliary fields/streets. Maximum projection of a CLSM stack with staining for anti‐acetylated alpha‐tubulin (green) and nuclei (blue). Abbreviations: cae, caecum; cf, ciliary field; cs, ciliary street; ec, ectocyst; en, endocyst; es, esophagus; ggl, cerebral ganglion; int, intestine; l, lophophore; p, peritoneum; ph, pharynx; pv, proventriculus; rm, retractor muscle.

**Figure 17:**
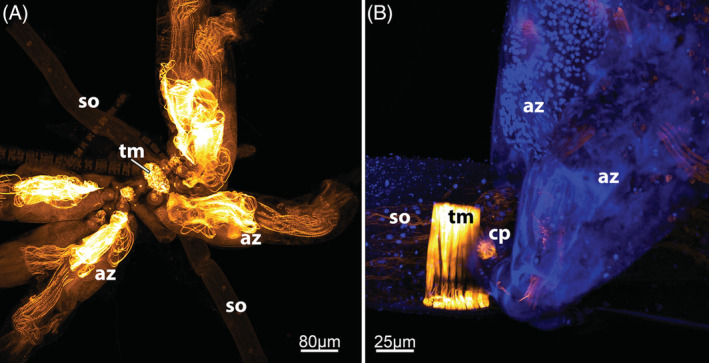
The transversal (‘dorso‐ventral’) muscle in the stolon in walkerioidean ctenostomes. (A) Group of autozooids of *Walkeria uva* on a stolon. The transversal muscle is seen in the main stolon as well as shorter side stolons. Maximum intensity projection, confocal laser scanning micrograph (CLSM) stack with staining for f‐actin (orange). (B) Lateral view of the transversal muscle in the stolon of *Mimosella* sp. Maximum projection, CLSM stack with staining for f‐actin (orange) and nuclei (blue). Note the f‐actin‐rich cells of the pore‐cell complex of the communication pore. Abbreviations: az, autozooid; cp, communication pore; so, stolon; tm, transversal muscle.

In phylactolaemates, it is known that bundles of cilia on the inner peritoneal layer facilitate circulation of the coelomic fluid, including its coelomocytes, within and between zooids (Mano, 1964; Mukai *et al*., 1997). Stenolaemata and Gymnolaemata are thought to lack this peritoneal ciliation except for the ctenostomes *Paludicella articulata* [Mukai *et al*. (1997); see also Weber *et al*. (2014); Fig. 16C, D], and *Hislopia malayensis* (Fig. 16A, B). In the latter, several ciliary bundles are present on the peritoneal layer of the foregut, mainly the pharynx, oesophagus and cardia. We suggest that peritoneal cilia are likely to be present in other ctenostomes, because in the absence of communication strands, fluid exchange between zooids is presumably maintained by ciliary beating. Contractile elements within the pore‐cell complexes, such as pore‐cell complex musculature in the ctenostome *H. malayensis* (Schwaha *et al*., 2011b), are also likely to be involved. In the ctenostome superfamily Walkerioidea an additional mechanism is present. Each stolonal element possesses a median transversal muscle, running from the basal to the frontal side (often refered to as ‘dorso‐ventral’; Ehlers, 1876; Franzén, 1960; Jebram, 1973*a*; Fig. 17). It is always situated in proximity to the feeding zooids and was observed to contract several times per minute (Jebram, 1973*a*). Consequently, there appear to be three different mechanisms involved in nutrient transport among ctenostomes: (*i*) circulation of coelomic fluid by ciliary peritoneal bundles (Paludicelloidea, Hislopioidea) assisted by contraction of pore‐cell complexes; (*ii*) circulation of coelomic fluid *via* activity of prominent transversal muscles (in stoloniferous Walkerioidea); and (*iii*) transport *via* funicular cords associated with pore‐cell complexes (Victorelloidea, Vesicularioidea and family Nolellidae of Arachnidioidea).

The colonial funicular connectivity of the Vesicularioidea, Victorelloidea and some Arachnidioidea (and possibly Benedeniporoidea; see Appendix S1, Table S1) probably evolved from an initial (proximal) funicular cord that acquired the ability to transport metabolites. Muscular elements reported for one vesicularioid ctenostome support the participation of the funiculus in food manipulation in the gut as observed in the cheilostome *Membranipora membranacea*. Similar functions could be inferred from the presence of a tubular, muscular proximal funiculus in phylactolaemates and cyclostomes (and also for the caecal ligament in cheilostomes). However, here it is attached to the cystid wall without connecting to a communication pore (Lutaud, 1962). In sexual zooids of Phylactolaemata and Cyclostomata the funiculus is almost always connected to spermatogenic tissue/testes that either develops directly on it or on the caecum. In gymnolaemates, spermatogenic tissue is sometimes developed on the funiculus, but more often on the cystid wall where it may be connected with the funiculus. In many ctenostomes the funiculus is connected with the ovary, suggesting a nutritive function during gametogenesis (Reed, 1991; Ostrovsky, 2013*a*).

It is noteworthy that the proximal funicular cord/caecal ligament is not connected to the rest of the ‘funicular system’ in the Cheilostomata, indicating that the anastomozing network of mesodermal cords originated independently of the original funiculus and should not be called a ‘funicular system’.

**Figure 18:**
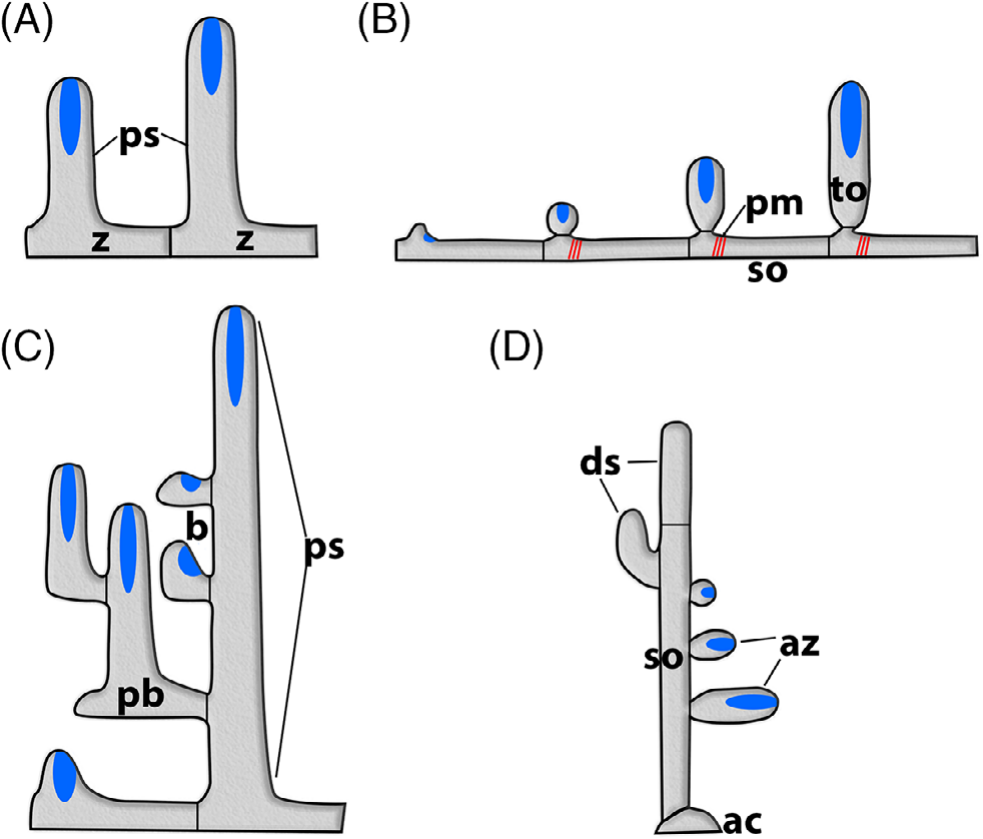
Examples of growth forms of selected ctenostome superfamilies with elongated peristomes and stolonate growth forms (redrawn and modified from Jebram, 1973*a*). The general outline of each zooid is shown in black, polypides and polypide buds are in blue (not to scale). Ontogenetically younger zooids or buds are shown on the left of A–C and at the top in D. (A) Simple colony type found for example in Nolellidae (Arachnidioidea) with zooids possessing elongated peristomes. (B) Stolonate colony type found in Walkerioidea. These ctenostomes have a creeping habit in which the proximal, creeping part is transformed into a stolon by the formation of a septum. A characteristic feature is a prominent transversal muscle that runs from the basal side to the frontal side of the stolon. (C) Victorelloidea also show elongated peristomes. One of their defining features is peristomial budding, i.e. buds are produced not only from areas attached to the substrate but also on the elongated peristome. (D) Vesicularoidea also form colonies with stolons. However, in contrast to Walkerioidea the stolons are not formed from creeping proximal parts of the original zooid but from the enlarged peristome. Abbreviations: ac, ancestrula; az, autozooid; b, buds; ds, developing stolons; pb, peristomial bud; pm, parietal musculature; ps, peristome; so, stolon; to, trophon; z, zooid.

The evolution of funicular interconnections between zooids clearly affects colonial integration and perhaps has influenced the diversity of these groups. However, two ctenostome superfamilies (Alcyonidioidea, Walkerioidea) without such connections show higher diversity with respect to their number of families and species than ctenostomes whose stolonal and autozooidal funicular elements are interconnected (Vesicularioidea, Victorelloidea, Nolellidae of Arachnidioidea) (Jebram, 1986*b*; Bock & Gordon, 2013). Thus, the evolution of funicular connectivity between zooids appears not to have a pivotal role in ctenostome diversification. Also, the structural and developmental complexity that have been argued to require physiological integration of the colony are present despite the lack of funicular connectivity between zooids. While Walkerioidea and Vesicularioidea form true stolons and thus have a somewhat similar colony arrangement, there is a crucial difference: Walkerioidea are primarily creeping forms with stolons (devoid of a stolonal funicular cord) derived from proximal portions of each zooid, whereas Vesicularioidea primarily form erect colonies with stolons (with a funicular cord) derived from peristomial tubes, i.e. elongations of the original apertural areas (Jebram, 1973*a*; Schwaha, 2019*a*; Fig. 18B, D). It is interesting that only species with elongated peristomes appear to possess colonial funicular connectivity: Victorelloidea, Vesicularoidea and the family Nolellidae of Arachnidioidea (Fig. 18A, C, D), and this could be investigated using different species of Arachnidiidae, which show high variations in peristome length. Some vesicularoideans which have secondarily developed a creeping colony form were described as lacking the funicular system within their stolons (Jebram, 1973*a*).

Another characteristic of interest is the presence of multiporous septula in some ctenostomes: the vast majority have a simple communication pore in interzooidal septa, but the genera *Pherusella* and *Flustrellidra* (without funicular connectivity) (Alcyonidioidea) and *Sundanella sibogae* (which may have funicular connectivity) (Victorelloidea) possess multiporous septula that are otherwise only known in Cheilostomata (Osburn, 1953; Marcus, 1941). It may be that these multiporous pore plates in ctenostomes evolved independently of those in cheilostomes.

A reticulate system of possibly funicular strands was described and depicted by Pergens (1889) in the ctenostome *Lobiancopora hyalina*, but this requires reinvestigation since more recent observations were not able to confirm these findings (see Hayward, 1985). The elaborate anastomosing mesodermal network connected with numerous communication pores found in Cheilostomata may represent a derived feature of this clade. This network allows rapid redistribution of energy resources throughout a colony and may have enabled the evolution of colonial ‘organs’ such as colonial growth zones, and highly developed morpho‐functional polymorphism by providing nutritional support to numerous non‐feeding specialized zooids (sexual, protective, sentry, etc.) (Lidgard *et al*., 2012). The inter‐ and intrazooidal funicular system also allows embryos to develop continuously in brood chambers during polypide recycling in placental species, thus enabling continuous larval production (Dyrynda & Ryland, 1982; Ostrovsky, 2013*a*,*b*).

It should be emphasized that many additional aspects of funicular evolution in Bryozoa remain to be uncovered by future studies.

#### (e) *Excretion and osmoregulation*

In bryozoans, excretion occurs mainly *via* coelomocytes that accumulate waste products within the trunk/visceral coelom (Harmer, 1891; Matricon, 1960; Mano, 1964; Bobin & Prenant, 1972; Gordon, 1977; Mukai *et al*., 1997). It was suggested that coelomocytes are expelled *via* the vestibular pore (Oka, 1895*a*,*b*; Mano, 1964; Schwaha *et al*., 2016) or *via* the forked canal in phylactolaemates (Verworn, 1887; Cori, 1890, 1893; Rogick, 1937). The forked canal was considered by some to be an excretory organ or even a vestigial metanephridium (Cori, 1890, 1893) while other authors disagreed (e.g. Braem, 1890; Oka, 1895*a*,*b*). The view that the forked canal functioned in excretion was largely based on observations of the phylactolaemate *Cristatella mucedo* where an excretory bladder is situated at the junction of the forked canal in the inner lophophoral concavity (Cori, 1890, 1893; Schwaha *et al*., 2011a). The bladder is commonly filled with several cells/coelomocytes that are thought to be expelled by its rupture (Verworn, 1887; Cori, 1890, 1893; Gerwerzhagen, 1913*b*). Accumulation of these cells occurs due to massive ciliation of the forked canal (Gruhl *et al*., 2009; Schwaha *et al*., 2011a); in some species cilia are reported to extend even into the basal part of median tentacles emanating from the forked canal (Marcus, 1934; Rogick, 1937; Schwaha, 2018). In addition, vital dye injected into specimens can be found in the forked canal and associated tentacles soon after injection (Marcus, 1934). These features are topologically and structurally similar to the densely ciliated metanephridial funnels of phoronids (Schmidt‐Rhaesa, 2007). In *Asajirella* and *Plumatella* an opening of the forked canal to the exterior is thought to function in coelomocyte disposal (Oka, 1895*b*; Malchow, 1978). However, a recent study on the genus *Plumatella* was not able to identify any such pore or gap in the basal membrane of the epithelial lining (Gruhl *et al*., 2009). Also, sperm have been found in the forked canal, suggesting a role in gamete release (Braem, 1890; T.F. Schwaha, personal observations). In some phylactolaemates peritoneal cells in the vicinity of the ciliated lining of the forked canal have a podocyte‐like arrangement (Gruhl *et al*., 2009).

Most coelomic lophotrochozoans including phoronids and brachiopods possess a blood vascular system that is essential for a metanephridial excretory system (Schmidt‐Rhaesa, 2007). It was previously assumed that the ancestor of bryozoans possessed a vascular system, which subsequently became reduced as a result of miniaturization of the individual zooids. The phylactolaemate forked canal may therefore represent a vestigial metanephridium due to its (*i*) topologically similar position to metanephridia of other lophophorates; (*ii*) dense ciliation as found in the metanephridial funnels; (*iii*) podocyte‐like arrangement of peritoneal cells close to the funnels; and (iv) function in excretion and gamete release. However, additional studies should investigate the ultrastructure and functional aspects of the forked canal on a broader scale among Phylactolaemata.

A comparable structure to the forked canal is not present in the circular lophophore of myolaemates. In Gymnolaemata, a supraneural coelomopore might represent a derived nephridiopore (Ostrovsky & Porter, 2011) and the intertentacular organ, positioned on the anal side with dense internal ciliation, might correspond to the phylactolaemate forked canal. An intertentacular organ is predominantly found in ancestrally broadcast‐spawning species and serves as an entrance for sperm and an exit for the release of fertilized oocytes (Ström, 1977; Reed, 1991; Temkin, 1994; Ostrovsky & Porter, 2011) as well as possibly coelomocytes or other excretory substances (Hincks, 1880). It is connected to an internal ciliated gutter in the broadcast‐spawning cheilostome *Membranipora serrilamella* (Hageman, 1981). Similar internal ciliated structures have been described in two brooding ctenostomes: *Alcyonidium polyoum* has an internal ciliated funnel (Matricon, 1963) and *Bowerbankia (Amathia) gracilis* has a pair of longitudinal internal ciliated ridges. In both species, these ciliated structures transfer fertilized oocytes to the brood chamber *via* a coelomopore (Reed, 1988). It was suggested that the ciliated gutter, funnel and longitudinal ridges are homologous (Reed, 1991).

Vital dye experiments showed that the tentacles probably play an important role in excretory processes, with their cells accumulating waste products that are subsequently discarded (Marcus, 1926*a*). An additional feature considered to relate to excretion was the formation of brown bodies (Ostroumoff, 1886; Harmer, 1891; Marcus, 1926*a*; Mukai *et al*., 1997). In many species, brown bodies are stored within the zooid after each polypide recycling (regeneration cycle) (e.g. Calvet, 1900; Cheetham & Cook, 1983; Boardman, 1998). In the most comprehensive review available, excretion was not considered the primary function of brown body formation (Gordon, 1977).

A peculiar statocyst‐like organ with a supposed excretory function is found only in the solitary and motile *Monobryozoon ambulans* (Remane, 1938; Gray, 1971). It consists of small, paired ciliated grooves situated laterally to the orifice. The grooves are filled with refractive concrements that appear to be rotated by ciliary activity and eventually expelled (Remane, 1938). It is not clear whether these organs are homologous to any other morphological structure, but it appears that they are infoldings of the vestibular wall.

Larval excretory organs are entirely unknown in Bryozoa (Zimmer & Woollacott, 1977*b*; Temkin & Zimmer, 2002). Other lophotrochozoan larvae (e.g. in phoronids, brachiopods, molluscs and annelids) usually possess protonephridia. In the cyphonautes larva, there is a slight indication of ciliary ‘tubules’ between the internal sac and digestive tract which could perhaps correspond to reduced protonephridia (Stricker, Reed & Zimmer, 1988). However, this character requires further assessment. Non‐feeding coronate larvae are characteristic of most Gymnolaemata and all Cyclostomata. Excretory systems appear to be absent in these larvae as well as in the mantle larva of Phylactolaemata. However, all these larvae can be considered as strongly modified and are of little use in reconstructing ancestral bryozoan larval features.

Nephridia in bilaterians are commonly regarded as excretory organs and their osmoregulatory function is overlooked. In some cases nephridia act in osmoregulation but not in excretion (e.g. Kamptozoa) (Emschermann, 1982). Studies of bryozoan osmolarity have not yet been conducted, but the absence of nephridial or other obviously osmoregulatory structures indicates that bryozoans are likely to be osmoconformers involving various tissues for homeostasis [see Gruhl & Bartoloameus (2008) concerning low‐osmolarity features of freshwater bryozoans].

#### (f) *Sexual reproduction*

Patterns of sexual reproduction in bryozoans are highly diverse. All bryozoans are colonial hermaphrodites and spermcasters with internal cross‐fertilization (intraovarian or near/post‐ovulatory) (Ostrovsky, 2008*a*), whose progeny are either spawned as zygotes or incubated with or without extraembryonic nutrition (EEN, e.g. Ostrovsky, Dick & Mawatari, 2007; Ostrovsky *et al*., 2006, 2009c; Ostrovsky, O'Dea & Rodgríguez, 2009a). Accordingly, either long‐lived planktotrophic or short‐lived endotrophic (matrotrophic or lecithotrophic) larvae develop [reviewed in Reed, 1991, Ostrovsky, Vávra & Porter, 2008, Ostrovsky, Gordon & Lidgard, 2009b and Ostrovsky (2008*b*, 2013*a*,*b*, 2019)].

In Phylactolaemata, hermaphroditic zooids produce many small oligolecithal oocytes. Fertilization probably takes place in the ovary and a single zygote is afterwards transferred to a brood sac where embryogenesis accompanied by matrotrophic nourishment occurs. A ring or terminal attachment structure develops during embryogenesis that is considered to be a placental analogue. The larva is a floating zooid/small colony with a ciliary hull (see Section II.1*b*) (Braem, 1897, 1908; Marcus, 1934).

Cyclostomes are viviparous. Female (or hermaphrodite) autozooids produce one or two small oligolecithal oocytes that, after fertilization in the ovary, are incubated in the coelom of the modified maternal zooid (gonozooid) with matrotrophic nourishment. The primary embryo buds numerous secondary embryos that multiply and grow (polyembryony) and are surrounded by a syncytial placental analogue – a modified membranous sac in the secondarily inflated maternal zooid (gonozooid). The ciliary larva is non‐feeding (Harmer, 1893, 1896, 1898; Borg, 1926; Nielsen, 1970; d'Hondt, 1977).

Phylactolaemata and Cyclostomata have highly specialized reproductive patterns, presumed characteristic for their entire groups. By contrast, gymnolaemates possess a variety of patterns, including broadcast spawning, brooding and viviparity. Several ctenostome taxa of the Alcyonidioidea, Hislopioidea and Walkerioidea as well as the early‐branching membraniporine cheilostomes produce numerous small oocytes that are shed into the water column after intracoelomic fertilization, where they develop into planktotrophic shelled larvae (cyphonautes) (Zimmer & Woollacott, 1977*a*; Wood, 2008; Nielsen & Worsaae, 2010). In almost all broadcast spawners, zygotes are released *via* the ciliated funnel of the intertentacular organ although some brooders also possess this organ (Ostrovsky & Porter, 2011). This pattern of sexual reproduction is considered ancestral for Gymnolaemata and possibly for all Bryozoa. The vast majority of gymnolaemates are brooders and have a topologically and functionally similar but structurally simpler supraneural pore which is also present in some ctenostome broadcast spawners (Ostrovsky, 2013*a*).

The remaining Gymnolaemata produce one to several meso‐ or macrolecithal eggs that are large in non‐placental brooders and either small or large when matrotrophic nutrition is present. At least five different patterns of embryonic incubation are known, either viviparous or brooding (Ostrovsky, 2019). In all of these, non‐feeding short‐lived larvae are formed which in few instances possess a non‐functional gut. The distribution of oogenetic modes, types of embryonic incubation and larval anatomy imply numerous independent shifts from a broadcast‐spawning pattern with a feeding larval stage to embryonic incubation with lecithotrophic larvae (Taylor, 1988; Reed, 1991; Ostrovsky, 2013*a*). Placentation has evolved independently many times among Gymnolaemata, and at least once in the Phylactolaemata and once in the Stenolaemata (Ostrovsky *et al*., 2009b, 2016; Ostrovsky, 2013*a*,*b*; Schwaha *et al*., 2019a). These changes were accompanied by shifts in oogenesis from oligolecithal to macrolecithal and occasionally by reversals back to oligolecithal oogenesis (Moosbrugger *et al*., 2012; Ostrovsky, 2013*a*,*b*; Nekliudova *et al*., 2019b).

### (3) Reconstructing ancestors

To reconstruct the ancestral bryozoan bauplan, an appropriate outgroup comparison is essential. Traditional scenarios (Farmer, 1977; Farmer, Valentine & Cowen, 1973) were based on the lophophorate concept emphasizing a sister relationship of bryozoans to phoronids (see also Mundy, Taylor & Thorpe, 1981). Due to the lack of preservation of soft tissues, the fossil record provides few clues concerning bryozoan origins and possible relationships to other phyla. Two older concepts are discussed in Appendix S2.

#### (a) *Common ancestral characters of bryozoans*

Since the appropriate outgroup remains unclear, assessing characters of bryozoans as apo‐ or plesiomorphic remains difficult, especially with regard to the character distribution of phylactolaemates and myolaemates. These two main bryozoan clades (see Fig. 2) show a dichotomous split of characters that render ancestral state reconstruction difficult. As set out in this review, these characters include: (*i*) a horseshoe‐shaped *versus* circular lophophore; (*ii*) an epistome *versus* no epistome; (*iii*) three coelomic canals versus one; (*iv*) kneading‐facilitated digestion *versus* rotation‐facilitated digestion; (*v*) a simple pharynx *versus* a myoepithelial suction pharynx; (*vi*) orthogonal body wall musculature *versus* modified body wall musculature; (*vii*) monomorphic *versus* polymorphic colonies; and (*viii*) an oral *versus* anal budding direction.

Whereas phylactolaemates are relatively well studied, there are still distinct gaps in our knowledge on myolaemate morphology. The latter particularly concerns soft‐body characters of cyclostomes that are necessary to the identification of ground‐pattern characters of this clade and ultimately for shared characters of all myolaemates.

Despite these gaps in our knowledge, some characters can be proposed for the last common bryozoan ancestor (LBA). Bryozoa is the sole invertebrate group consisting entirely of colonial animals. There are only a very few solitary ctenostomes (e.g. monobryozoids) that are thought to have secondarily lost their colonial habit due to adaptation to a mesopsammal (interstitial spaces in marine sand) life or to deep‐sea habitats (see Ott & Schwaha, 2020; Schwaha *et al*., 2019b). Some cystid appendages of aethozoid ctenostomes are kenozooidal and indicate their colonial origin (Schwaha *et al*., 2019b). Coloniality is thus a key feature of the LBA that has strongly influenced aspects of their structure, physiology and development. The production of asexual buds to facilitate coloniality would have occurred on three sites in the LBA: one distal and two lateral. This ‘cruciform’ pattern is present in early‐branching Phylactolaemata, Cyclostomata and Gymnolaemata [see Schwaha *et al*. (2016) for a recent review; for budding in Gymnolaemata see d'Hondt (1982, 1983) and Nikulina (2002); for Cyclostomata see Borg (1926), Harmelin (1976), Ostrovsky & Taylor (1996) and Ostrovsky (1998)]. Since polypides are formed prior to the cystid in Phylactolaemata and the myolaemate cyclostomes, this character is considered an ancestral feature of the LBA.

Besides coloniality, another apomorphic character is the retraction of the polypide *via* prominent retractor muscles. These retractor muscles were previously considered derivatives of the original longitudinal body wall musculature (Jebram, 1986*a*), implying that body wall musculature was present in the ancestral bauplan. This is supported by its presence in the early‐branching Phylactolaemata and its derivates in the myolaemate Cyclostomata (see Section II.1*d*) and in worm‐shaped lophotrochozoans (see Schwaha & Wanninger, 2012). While polypide retraction is caused by contraction of the retractor muscles, protrusion including eversion of the tentacle sheath is achieved mainly *via* contraction of the circular (and possibly diagonal) body wall musculature in phylactolaemates (Gawin *et al*., 2017). Protrusion of the polypide *via* annular muscles in stenolaemates and *via* the parietal muscles in gymnolaemates evolved independently within the two clades of Myolaemata.

Additional features that can be proposed for the LBA are: (*i*) two longitudinal tentacle muscle bands, present in all recent bryozoans (Gawin *et al*., 2017; Schwaha & Wanninger, 2018; Schwaha *et al*., 2018); (*ii*) a subepithelial cerebral ganglion with a circum‐oral nerve ring; (*iii*) probably six tentacle neurite bundles, three frontal and three abfrontal [Schwaha (2019b) and Section II.2*c*); (*iv*) a U‐shaped gut (see Section II.2*b*); and (*v*) a coelomic cavity with probably at least one canal at the lophophoral base; only the ring canal is present in all bryozoans (see characters P2 and M4 in Sections II.1*b* and II.1*c*, respectively).

The body cavity of all bryozoans has coelomopores used for the release of gametes and possibly also coelomocytes. Pores at the tentacle tips and the lophophoral base are widely distributed and can be considered as part of the LBA. These pores are necessary due to the lack of a nephridial system, which in other coelomate organisms allows the release of substances or gametes from the body cavity. The forked canal of phylactolaemates, which was previously considered to represent a vestigial metanephridium, and the topologically similar intertentacular organ of gymnolaemates (see Section II.2*e*) indicate that a ciliated tubular structure was probably present on the anal side of the lophophoral base in the LBA.

The LBA likely had numerous small oligolecithal oocytes that were fertilized intracoelomically. Zygotes were spawned *via* a coelomopore and developed into larvae in the water column (see Ostrovsky, 2013*a*, 2019). Strong similarities in the structure of cyphonautes larvae compared to heterogenous coronate larvae imply that the ancestral larval type, of at least the Gymnolaemata, was planktotrophic (see Nielsen & Worsaae, 2010), whereas lecithotrophic or planktotrophic larvae are equally possible in the LBA.

#### (b) *Diversification of bryozoan clades*

The invasion of fresh water resulted in the origin of the Phylactolaemata. This transition from marine to freshwater habitats probably took place in the Palaeozoic; the earliest fossils of statoblasts are from the Triassic (Kohring & Pint, 2005) or even the Permian (Vinogradov, 1996). It is not known if the zooidal size of ancestral bryozoans was similar to that of recent phylactolaemates, but the size of fossil statoblasts (often 0.8–1 mm; see Kohring & Pint, 2005) indicates that at least those extinct forms were of similar size to many recent ones. Larger zooidal sizes in some recent phylactolaemates is correlated with a horseshoe‐shaped lophophore; secondarily reduced circular forms are only found in the small Fredericellidae (see Section II.1*b*, character P1). An oral budding direction is the dominant mode in most Phylactolaemata (see Jebram, 1973*b*).

The earliest myolaemate (calcified stenolaemate) fossils date back to the early Ordovician, and it is generally accepted that calcification evolved from soft‐bodied ancestors (Ernst & Schäfer, 2006), which have poor preservation potential. The presence of fossil ctenostomes in the Paleozoic indicates that the myolaemate clades diverged at least by the early Paleozoic. All myolaemates show a myoepithelial pharynx and a truly circular lophophore. These characters were accompanied by modifications of the cuticle and body wall, and the associated musculature, into a more protective and economic design (Jebram, 1986*a*). This lineage shifted predominantly to an anal budding direction although conserving the potential for budding on either side.

## III. KEY NOVELTIES IN BRYOZOAN EVOLUTION

Modification of the bryozoan ancestral ground plan involved a number of important morphological innovations. Among the key novelties in the evolution of Phylactolaemata were the acquirement of statoblasts and matrotrophic embryonic brooding, including a heterochronic shift of asexual development into the larval phase. The innovation of statoblasts allowed this clade to live in ephemeral water bodies and with a highly efficient dispersal system. Placentation allowed the development of larger larvae, possibly with more rapid development, which could both have important ecological implications for successful survival in epibiotic communities (Ostrovsky *et al*., 2009b; Ostrovsky, 2013*b*). A heterochronic shift of asexual budding into the larval stage reduced the vulnerability of the larval stage owing to shorter duration of embryonic brooding of this specialized larva, potentially enabling faster occupation of available niches. An important feature of many phylactolaemate taxa is that they are motile for at least some part of their life cycle (Schwaha *et al*., 2016).

A key novelty within the Myolaemata was calcification of the body wall, which evolved independently at least two or three times, resulting in the origin of Stenolaemata and Cheilostomata [reviewed in Ernst & Schäfer (2006), Taylor & Waeschenbach (2015), Taylor *et al*. (2015b); for skeletal novelties see Jablonski, Lidgard & Taylor (1997)]. The resulting loss in flexibility of the body wall led to a transition to different hydrostatic mechanism(s) accompanied by rearrangement of the body wall musculature. The increased robustness provided by calcified cystids enabled new colony forms. The diversity of non‐calcified forms in Phylactolaemata and ‘Ctenostomata’ is comparatively poor with predominantly encrusting forms, whereas calcified forms adopt a variety of upright and highly complex colony structures [see e.g. Hageman (2003) for diversity of colonial architecture].

Among Stenolaemata calcification of the body wall led to the formation of a membranous sac with ring muscles (see Section II.1*d*, character S2). Stenolaemates acquired matrotrophic viviparity inside enlarged gonozooids that could have triggered the evolution of polyembryony (Ostrovsky, 2013*a*,*b*). Despite the paradoxical nature of releasing genetically identical embryos (Ryland, 1996; Craig *et al*., 1997; Hughes *et al*., 2005; Jenkins *et al*., 2017) this reproductive pattern appears successful and may be correlated with the Mesozoic radiation of Cyclostomata.

In the Gymnolaemata, the presence of parietal muscles in ctenostomes shows that they were acquired before calcification in Cheilostomata. Cheilostome evolution was accompanied by the origin of a complex funicular system providing increased colonial integration (see Section II.2*d*). This is evident in the appearance of non‐feeding polymorphs, supplied by feeding autozooids. Polymorphic kenozooids present on the frontal wall increased protection of the colony, followed by the acquisition of protective frontal shields of various morphologies (Gordon, 2000; Gordon & Voigt, 1996; Lidgard *et al*., 2012). Finally, highly complex morpho‐functional polymorphism led to the appearance of so‐called cormidial structures – zooidal complexes consisting of autozooids with adventitious avicularia and kenozooids forming their frontal shields, as well as the protective brood chamber (Lidgard *et al*., 2012; Schack *et al*., 2019). Such polymorphism evolved independently in all three main lineages of Myolaemata but is absent in Phylactolaemata.

Pore‐cell complexes of Gymnolaemata are involved in the transport and distribution of metabolites within the colony (see Bobin, 1977; Mukai *et al*., 1997) and thus constitute an important precursor to polymorphism. They occur in both ‘Ctenostomata’ and Cheilostomata. Further modification of the communication system led to the origin of multiporous septula in cheilostomes along with a complex branching ‘funicular’ network (see Cheetham & Cook, 1983). We consider the evolution of this network as a key novelty of cheilostomes that enhanced colonial integration and metabolite transfer.

Additional innovations in Gymnolaemata were the protective collar and operculum for closing the zooidal aperture, and embryonic incubation chambers. The vast majority of gymnolaemates incubate their progeny, and a variety of methods and accessory structures, including placentation, have evolved independently in this group (Ostrovsky, 2013*a*,*b*). Embryonic incubation was associated with a shift from an oligolecithal to macrolecithal mode of oogenesis, resulting in the evolution of non‐feeding larvae, possibly triggering the evolutionary radiations of bryozoans seen in the fossil record (Taylor, 1988). This change of mode of oogenesis is present in all three major clades (Phylactolaemata, Stenolaemata and Gymnolaemata) (Ostrovsky, 2013*a*,*b*, 2019).

## IV. CONCLUSIONS

(1) This review represents the first attempt to assess morphological characters of bryozoan soft tissues in a phylogenetic context and to assign these characters to the topology of the most recent molecular trees.

(2) Many character states are likely to be improved by future analyses, especially since the morphological diversity and variability of the different organ systems in both Phylactolaemata and Myolaemata is poorly known.

(3) Phylogenetic approaches will ultimately aid in determining the sister group of bryozoans and thus allow a detailed reconstruction of ancestral characters of bryozoans. In addition, new phylogenomic data on the three large bryozoan clades will yield a better understanding of the distribution of colonial and zooidal traits in each group.

## Supporting information


**Appendix S1.** Comparative analysis of funicular structure in ctenostome bryozoans.

**Appendix S2.** Two older views on the potential evolutionary origins of Bryozoa.

**Table S1.** Funicular variation in Gymnolaemata.
