# Supplementary material for: Key novelties in the evolution of the aquatic colonial phylum Bryozoa: evidence from soft body morphology
Source: Biol Rev Camb Philos Soc. 2020 Feb 7;95(3):696–729. doi: 10.1111/brv.12583 (PMC7317743; doi:10.1111/brv.12583)
Supplement: Supplementary file 1 — Appendix S1. Comparative analysis of funicular structure in ctenostome bryozoans. [file BRV-95-696-s001.docx]

**Appendix S1. Comparative analysis of funicular structure in ctenostome bryozoans.**

The below text refers to the main superfamilies proposed by Todd (2000): Paludicelloidea, Alcyonidioidea, Hislopioidea, Arachnidioidea, Walkerioidea, Victorelloidea and Vesicularioidea.

**(1) Paludicelloidea**

*Paludicella articulata* (superfamily Paludicelloidea) possesses two funicular cords (Hancock, 1850; Allman, 1856; Kraepelin, 1887; Braem, 1890, Fig. 14A, C). One emanates from the posterior end of the caecum (i.e. at the proximal side of the zooid hence proximal funiculus) and a second emanates more anteriorly from the caecum, close to the pylorus (accordingly, distal funiculus). Both of these funiculi have longitudinal musculature and both run to the body wall (proximal to the testis, and distal to the ovary in sexual zooids). Consequently, the posterior funiculus resembles the condition found in the Phylactolaemata and Cyclostomata.

Together with the family Paludicellidae (one genus, one or two species), the Panolicellidae (one genus, one or two species) have previously been assigned to the Paludicelloidea (Jebram, 1986*b*; Bock & Gordon, 2013). The latter family displays a very different colony morphology than the Paludicellidae and its association with the superfamily is only based on its ontological sequence of muscle development (Jebram, 1985, 1986*b*; see also Vieira *et al*., 2014). *Panolicella* differs greatly from *Paludicella* in its zooidal morphology with elongated peristomes and in its colony structure, and its placement among Paludicelloidea is doubtful. *Panolicella* also shares several characters with *Nolella* (Arachnidioidea) and *Victorella* (Victorelloidea) (Jebram, 1985; Vieira *et al*., 2014). The structure of the funicular system is not clear in *Panolicella*, but seems to be a cord associated with the ovary coming from the caecum tip and perhaps runs to the thin wall in the elongated basal parts of the cystid (Jebram, 1985).

**(2) Alcyonidioidea**

In the superfamily Alcyonidioidea (five families, ~18 genera, ~103 species) the funiculus runs from the caecum tip to the lateral cystid wall in a similar fashion to the proximal funiculus of *Paludicella* (Gordon, 1975*c*). In some species a second (anterior or distal) funicular cord is present in autozooids, running in parallel to the posterior funiculus to the cystid wall from the stomach area close to the pylorus. Muscular fibres were reported in the funiculus of some species (Prouho, 1892; Pace, 1906; Matricon in Lutaud, 1962).

**(3) Hislopioidea**

The Hislopioidea (one family, three genera, eight or nine species) form runner-like, encrusting colonies similar to those of most Alcyonidioidea. A funiculus was reported as absent by Annandale (1911), but at least one species (*Hislopia malayensis*) possesses a distal funiculus from the pyloric area to the lateral body wall (T.F. Schwaha, unpublished data). However, no musculature is present (Schwaha *et al*., 2011*b*).

**(4) Arachnidioidea**

The superfamily Arachnidioidea (~three families, ~11 genera, ~50 species) comprises three families (Arachnidiidae, Nolellidae and Immergentidae) and sometimes the unusual solitary Monobryozoidae (whose phylogenetic affinities remain dubious). In general, the funicular system is considered as reduced in the Arachnidioidea (Jebram, 1973*a*). This assumption was clearly biased by the expectation that funicular cords connect the polypide with communication pores which now appears to be invalid. An absence of the funiculus has also been stated for the name-giving Arachnidiidae (Annandale, 1916). Only in the Nolellidae has a funicular cord from the caecum to the communication pore, and thus allowing interzooidal communication, been reported (Calvet, 1900; Franzén, 1960). Muscular fibres associated with the funiculus have so far been reported only for one species of *Nolella*, and the insertion area of the funiculus remains unclear (Schwaha & Wanninger, 2018).

**(5) Walkerioidea**

In the Walkerioidea (eight families, ~11 genera, ~33 species) the single funiculus runs from the proximal caecum tip and either attaches to the lateral body wall or approaches the interzooidal septum. For some species, it is still not clear whether the funicular cord joins the pore-cell complex or attaches to the cystid wall close to it, since both variants are depicted in the literature (van Beneden, 1845*b*; Ehlers, 1876; Marcus, 1926*a*; Banta, 1968). Possibly both forms exist, one attached to the pore and the other attached near to it. Funicular cords are absent in the elongated, primarily creeping, stolons which interconnect zooids within colonies of this superfamily (Jebram, 1973*a*). Muscle fibres in the funiculus of autozooids were identified or at least indicated in three species/genera: *Farrella repens* (van Beneden, 1845*b*), *Triticella minini* (Grischenko & Chernyshev, 2015) and *Mimosella* sp. (Schwaha, 2019*a*). In the latter genus, two funicular muscles similar to those of *Paludicella* were observed. Mimosellidae also have a flexor muscle in the proximal part of each autozooid just above the pore plate. This muscle runs from a less-rigid proximal part of the cuticle to lateral slightly more distal parts and acts to bend the zooids towards the substrate (Silén, 1950). Given its similar orientation to funicular structures in other genera, this muscle may have evolved from a funiculus. An alternative origin for these bending muscles could be parietal muscles.

**(6) Vesicularioidea and Victorelloidea**

In the superfamilies Vesicularioidea (~four families, ~10 genera, ~ 96 species) and some Victorelloidea (three families, six genera, 12 species) a single funiculus runs from the caecum tip to the pore-cell complex of the septal pore and connects it to the funicular cord of the neighbouring autozooid or stolon (Müller, 1860; Reichert, 1870; Joliet, 1877; Calvet, 1900; Zirpolo, 1933; Brien & Huysmans, 1937; Braem, 1951; Reed, 1988; Smith *et al*., 2003). This has become the most common description of the ‘ctenostome’ funicular system but actually structurally and physiologically represents the most complex variant in ctenostomes. Both zooidal and stolon funicular cords have a central lumen (see evidence for the vesicularioid *Amathia verticillata* in Carle & Ruppert, 1983). Muscles have been reported in a caecal ligament resembling a funicular cord in *A. lendigera* (Matricon in Lutaud, 1962). In some species from both superfamilies the proximal funiculus can split, with an additional branch running to the ovary on the lateral cystid wall.

**(7) Additional remarks**

Two funicular cords, anterior and posterior, emanating from the caecum and attaching to the lateral body wall are present in *Pottsiella erecta* (Annandale, 1911; Braem, 1940*b*), which resembles the situation in some Paludicelloidea and Alcyonidioidea. Some vesicularoideans appear to have reduced their stolonal funicular system, which might be a result of the change in colonial morphology (Jebram, 1973*b*).

Data on the ctenostome funicular system are scant, incomplete and sometimes controversial, making it in many cases impossible to understand its structure using published descriptions and schemes. This feature also has been commonly neglected, and ethanol fixations might have caused these fragile thread-like structures to collapse or become undiscernable. For example, the funicular system in the ctenostome superfamily Benedeniporoidea remains almost unknown.
