# Supplementary material for: Key novelties in the evolution of the aquatic colonial phylum Bryozoa: evidence from soft body morphology
Source: Biol Rev Camb Philos Soc. 2020 Feb 7;95(3):696–729. doi: 10.1111/brv.12583 (PMC7317743; doi:10.1111/brv.12583)
Supplement: Supplementary file 2 — Appendix S2. Two older views on the potential evolutionary origins of Bryozoa. [file BRV-95-696-s002.docx]

**Appendix S2. Two older views on the potential evolutionary origins of Bryozoa**

**(1) Sister-group to Phoronida**

The lophophorate concept unites Bryozoa with Phoronida and Brachiopoda, with Bryozoa as sister to phoronids (reviewed in Hyman, 1959; Farmer *et al*., 1973; Ostrovsky, 2013*a*). Anatomical similarities, the presence of asexual budding and the ability to retract the anterior part of the body were among the characters used to support this scenario (Farmer, 1977).

The boring phoronid *Phoronis ovalis* shares several features with bryozoans: (1) coloniality (perhaps only temporary) due to asexual budding; (2) an infundibuliform valve resembling the cardiac valve of bryozoans present at the end of the oesophagus (Du Bois-Reymond Marcus, 1949; Lönöy, 1954); (3) a partially retractable tentacle crown and anterior body (Harmer, 1917; Brattström, 1943). Recently, new data on the nervous systems of the three lophophorate taxa showed similarities among these three phyla (Temereva, 2017*a*,*b*; Temereva & Tsitrin, 2015; Temereva & Kosevich, 2016).

**(2) Sister-group to Kamptozoa**

An alternative concept was that Bryozoa is sister to Kamptozoa. From a morphological perspective, adult specimens of the two phyla show only superficial resemblances such as a ciliated tentacle crown. Larval features including metamorphosis were once considered similar among these two phyla (e.g. Nielsen, 1971), but are now known to be insufficient to support a sister-group relationship (Nielsen, 2012). In their adult morphology, the following differences are present between these two phyla: (1) Bryozoa are coelomate whereas Kamptozoa are acoelomate; (2) early-branching Kamptozoa are solitary whereas Bryozoa are colonial; (3) there is a downstream food-collecting system in Kamptozoa *versus* an upstream food-collecting system in Bryozoa (due to the different arrangement of ciliation of the tentacles); and (4) the anus opens outside the tentacle crown in Bryozoa *versus* within the crown in Kamptozoa. Only a few molecular phylogenies have supported a sister-group relationship of bryozoans to kamptozoans (e.g. Helmkampf *et al*., 2008) or combined them with the enigmatic Cycliophora as ‘Polyzoa’ (Hejnol *et al*., 2009). [It should be noted that the term ‘Polyzoa’ has been a synonym (nowadays rejected) for Bryozoa and was used regularly until the late 20th century (e.g. Hastings, 1979)]. The most recent phylogenies supported kamptozoans as the sister group to molluscs as ‘Tetraneuralia’ (Wanninger, 2009, Marlétaz *et al*., 2019).
