# Supplementary material for: Key novelties in the evolution of the aquatic colonial phylum Bryozoa: evidence from soft body morphology
Source: Biol Rev Camb Philos Soc. 2020 Feb 7;95(3):696–729. doi: 10.1111/brv.12583 (PMC7317743; doi:10.1111/brv.12583)
Supplement: Supplementary file 3 — Table S1. Funicular variation in Gymnolaemata. [file BRV-95-696-s003.docx]

**Table S1.** Funicular variation in Gymnolaemata.

| **Taxon** | **Number of zooidal funiculi** | | **Zooidal funiculus runs** | | **Zooidal funiculus** | **Muscular elements in funiculus** | **Funicular system in stolons** | **Connection of zooidal funiculus with gonad(s)**  **(where present)** | **References** |
| --- | --- | --- | --- | --- | --- | --- | --- | --- | --- |
|  | **single** | **pair** | **from** | **to** | **simple or**  **branching** | **presence / absence** | **presence / absence** | **presence/absence** |  |
| **CTENOSTOMATA**  **Alcyonidioidea**  *Alcyonidium albidum* | + | ‒ | caecum (area close to pylorus) | zooidal wall | simple |  |  | +  ovary on funiculus | Prouho (1892) |
| *Alcyonidium polyoum* | + | ‒ | caecum tip | zooidal wall | simple | + |  | +  ovary on caecum | Matricon (1963);  Matricon in Lutaud (1962) |
| *Alcyonidium gelatinosum* | + | ‒ | caecum tip | zooidal wall | simple | + |  | +  ovary on caecum | Faulkner (1933);  Chrétíen (1957); T.F. Schwaha (unpublished data) |
| *Alcyonidium hirsutum* | + | ‒ | caecum | zooidal wall | simple |  |  | +  ovary is “in association with the funiculus of the caecum” | Owrid & Ryland (1991, p. 319) |
| *Alcyonidium duplex* | ‒ | + | (1) caecum tip  (2) pylorus | (1) zooidal wall  (2) zooidal wall | simple  simple | ?^1^ |  | +  ovary on caecum | Prouho (1892) |
| *Alcyonidium variegatum* | ‒ | +^2^ | caecum | zooidal wall | simple | ?^1^ |  |  | Prouho (1892) |
| *Lobiancopora hyalina*  (dense funicular network ?)^3^ |  |  |  |  | branching? |  |  |  | Pergens (1889); Hayward (1985) |
| *Flustrellidra hispida* | + | ‒ | caecum tip | zooidal wall | simple | + |  | +  ovary on funiculus | Prouho (1892);  Pace (1906);  Matricon in Lutaud (1962) |
| *Pherusella tubulosa* | ‒ | +^4^ | caecum | zooidal wall | simple |  |  |  | Prouho (1892) |
| **Paludicelloidea**  *Paludicella articulata* | ‒ | + | (1) caecum tip  (2) pylorus | (1) zooidal wall  (2) zooidal wall | simple  simple | +  + |  | +  gonads on zooidal wall^5^ | Allman (1856);  Kraepelin (1887); Braem (1890); Weber *et al*. (2014); Schwaha & Wanninger (2018) |
| **Hislopioidea**  *Hislopia malayensis* | + | ‒ | caecum (area close to pylorus) | zooidal wall | simple | ‒ |  | –  ovary on zooidal wall | T.F. Schwaha (unpublished data) |
| **Benedeniporoidea**  *Benedenipora catenata* | each zooid has a longitudinal cord that approaches communication pores in the distal and proximal zooidal walls^6^ | | | | simple |  |  |  | Pergens (1889) |
| *Protobenedenipora labiostomelloides* | +? | ‒ | ? | ? | simple |  |  |  | D’Hondt & Schopf (1984) |
| **Arachnidioidea**  *Monobryozoon ambulans* | + | ‒ | caecum | zooidal wall | simple | + | n/a | ‒  ovary on caecum | Gray (1971) |
| *Aethozooides uraniae* | + | – | caecum | zooidal wall | simple | – |  | – ovary on body wall | Schwaha *et al*. (2019*a*) |
| *Cryptoarachnidium argilla* | + | ‒ | caecum | ? | simple |  |  |  | Banta (1967);  Jebram (1986*b*) |
| *Franzenella limicola* | + | ‒ | caecum tip | communi-cation pore in septum between zooids | simple? |  |  | +  ovary on funiculus | Franzén (1960) |
| *Nolella dilatata* | ‒ | +^7^ | caecum tip | communi-cation pore in septum between zooids |  |  |  | +  ovary on caecum | Calvet (1900) |
| *Nolella blakei* |  |  | caecum tip |  |  |  |  |  | Rogick (1949) |
| *Nolella* sp. | +? | - | caecum tip | ? | simple | + |  | ovary on zooidal wall | Schwaha & Wanninger (2018) |
| **Walkerioidea**  *Mimosella verticillata* | + | ‒ | caecum tip |  | simple |  | – |  | Hincks (1862, 1880) |
| *Farella repens* | + | ‒ | caecum tip | septal area between zooid and stolon^8^? | simple | ? | – | +  testis on funiculus,  ovary on zooidal wall | van Beneden (1845*b*); Hincks (1880); Marcus (1926*a*); Jebram (1973*a*); Hayward (1985) |
| *Triticella minini* | + | ‒ | caecum tip | zooidal wall | simple | + |  |  | Grischenko & Chernyshev (2015) |
| *Triticella flava* | + | ‒ | caecum tip | zooidal wall^9^ | simple |  | ‒ | gonads on zooidal wall | Reverter-Gil *et al*. (2016); Ström (1969) |
| *Triticella calveti* | + | ‒ | caecum tip | zooidal wall^9^ | simple |  | ‒ |  | Hayward (1978) |
| *Triticella pedicellata* | + | ‒ | caecum tip | zooidal wall^9^ | simple |  | ‒ |  | Hayward (1985) |
| *Triticella gracilis* | + | ‒ | caecum tip | zooidal wall^9^ | simple |  | ‒ |  | d’Hondt & Hayward (1981) |
| *Hypophorella expansa* | + | ‒ | caecum tip | zooidal wall | simple | - | ‒ | ‒  gonads on zooidal wall | Ehlers (1876); Pröts *et al*. (2019) |
| *Bathyalozoon foresti* | + | ‒ | caecum tip | zooidal wall | simple |  | ‒ |  | d’Hondt (1976) |
| *Bantariella cookae* | + | ‒ | caecum | communi-cation pore in septum between zooid and stolon | simple |  | ‒ |  | Banta (1968) |
| *Mimosella bigeminata* | + | ‒ | caecum tip |  | simple |  | ‒ |  | Waters (1914) |
| *Mimosella* sp. | - | + | (1) caecum tip  (2) pylorus | zooidal wall | simple | + | - | ? | Schwaha (2019*a*) |
| **Victorelloidea**  *Victorella pavida* | ‒ | +^10^ | caecum tip | horizontal funicular cord or  communi-cation pore in septum between zooids | branching | + |  | +  gonads on zooidal wall | Hincks (1880); Kraepelin (1887); Braem (1951); Soule (1957); Hayward (1985); Jebram (1986*b*); Annandale (1911) |
| *Victorella continentalis* | ‒ | +^11^ | caecum | horizontal funicular cord |  |  |  |  | Braem (1951) |
| *Victorella symbiotica* |  |  | caecum tip |  |  |  |  |  | Braem (1951) |
| *Tanganella muelleri* | ‒ | +^12^ | caecum (area close to pylorus) | communi-cation pore in septum between zooids | branching |  |  | +  gonads on zooidal wall | Braem (1951) |
| *Bulbella abscondita* | ‒ | +^13^ | caecum tip | communi-cation pore in septum between zooids or between zooid and stolon |  |  |  | ovary on zooidal wall | Braem (1951);  Hayward (1985) |
| *Sundanella sibogae* | ‒ | + | caecum tip | horizontal funicular cord or  communi-cation pore in septum between zooids |  |  |  |  | Harmer (1915);  Marcus (1941) |
| *Pottsiella erecta* | +^14^ | ‒ | caecum tip | communi-cation pore in septum between zooids (?) | branching |  |  | +  gonads on zooidal wall | Braem (1940*b*);  Smith *et al*. (2003) |
| **Vesicularioidea**  *Amathia gracilis* | + | +^15^ | caecum tip | communi-cation pore in septum between zooid and stolon | simple, and branching^15^ |  | + | –  ovary on zooidal wall | Braem (1951);  Jebram (1973*b*);  Reed (1988) |
| *Amathia pustulosa* | + | ‒ | caecum tip | communi-cation pore in septum between zooid and stolon | simple |  | + |  | Calvet (1900); Brien & Huysmans (1937) |
| *Amathia imbricata* | + | ‒ | caecum | communi-cation pore in septum between zooid and stolon | simple |  | + |  | Joliet (1877);  Bobin (1958, 1964, 1971, 1977);  Reverter-Gil *et al*. (2016) |
| *Amathia imbricata* | + | – | caecum | lateral body wall | simple | ? |  |  | Wiese *et al*. (1980) |
| *Amathia semiconvoluta* |  |  |  |  |  |  | + |  | Calvet (1900) |
| *Amathia verticillata* | + | ‒ | caecum tip | communi-cation pore in septum between zooid and stolon | simple |  | + | ovary on zooidal wall | Müller (1860); Zirpolo (1933); Carle & Ruppert (1983) |
| *Amathia lendigera* | + | ‒ |  |  |  | + | + |  | Waters (1914);  Matricon in Lutaud (1962) |
| *Vesicularia spinosa* | + | ‒ | caecum tip | communi-cation pore in septum between zooid and stolon | simple |  | + |  | van Beneden (1845*a*; Bobin (1958) |
| *Cryptopolyzoon wilsoni* | + | ‒ | caecum tip |  |  |  |  |  | Dendy (1888) |
| *Terebripora comma* |  |  |  |  |  |  | + |  | Bobin & Prenant (1954) |
| **species of uncertain affinity** |  |  |  |  |  |  |  |  |  |
| *Labiostomella gisleni* | + | ‒ | caecum tip | communi-cation pore in proximal zooidal wall | simple |  |  |  | Silén (1944*b*) |
| *Panolicella nutans* |  |  | caecum tip | ? |  |  |  | +  ovary on caecum | Ryland (1958);  Kayser (1984); Jebram (1985) |
| **CHEILOSTOMATA**  (complex funicular network, connected with communication pores)  **Malacostegina**  *Electra pilosa* | ‒ | + | (1) caecum  (2) pylorus | (1) zooidal wall  (2) zooidal wall | simple  simple | +  + |  | ‒  gonads on zooidal wall | T.F. Schwaha (unpublished data) |
| *Membranipora membranacea* | + | ‒ | caecum | zooidal wall | simple | + |  | ‒  gonads on zooidal wall | Lutaud (1962) |
| **Neocheilostomina**  13 spp. | + | ‒ | caecum | zooidal wall | simple | +^16^ |  | ‒  gonads are predominantly on zooidal wall | T.F. Schwaha (unpublished data) |

^1^ In *Alcyonidium duplex* and *A. variegatum* the funiculi have been depicted schematically as muscle-like threads (Prouho, 1892).

^2^ In *A. variegatum* the funiculus has been depicted schematically consisting of five threads in two groups (3+2), which are attached separately to the left side and to the tip of the caecum (Prouho, 1892).

^3^ In *Lobiancopora hyalina* it is unknown whether there is connection of the supposed funicular network with the polypide (Pergens, 1889; Hayward, 1985).

^4^ In *Pherusella tubulosa* the funiculus has been depicted schematically as two groups (~3 proximal + 1 distal thread) attached to the right side of the caecum (Prouho, 1892).

^5^ In *Paludicella articulata* the proximal funiculus (connected with the caecum tip) runs proximally to the spermatogenic tissue on the cystid wall, and the distal funiculus (connected with the pylorus) runs laterally to the ovary on the cystid wall.

^6^ In *Benedenipora catenata* it is unknown if there is connection of the funicular network and polypide.

^7^ According to Calvet (1900) there are two funicular cords in zooids of *Nolella dilatata*: a proximal cord in the peristome connects the caecum tip with a pore plate in the basal part of the zooid, whereas a horizontal cord connects the distal and proximal pores. These cords do not fuse together. Cord-like structures in cystid appendages that approach pore plates and interconnect neighbouring zooids were depicted but not described in *N. blakei* (Rogick, 1949). If those drawings are correct then the situation described by Calvet (1900) could be common for *Nolella* in general.

^8^ In *Farella repens* the proximal attachment of the funiculus was depicted by van Beneden (1845*b*) above the septum.

^9^ In *Triticella pedicellata* and *T. gracilis* the septum, which separates the distal part of the zooid and its peduncle, is formed in older zooids that eventually regenerate the distal part. In this respect, it is more probable that the funiculus is attached to the zooidal wall above or below the septum and even may pass through it. This view is supported by drawings of *T.* *calveti* in Hayward (1978) and *T. flava* in Reverter-Gil *et al*. (2016).

^10^ In *Victorella pavida* a proximal funicular cord runs from the caecum tip and connects it with either a septal pore in the basal part of the zooid or fuses with a basal cord which connects to neighbouring zooids (Soule, 1957). Since there are four septae in each zooid, the basal cord theoretically should branch to all septal pores. The proximal cord sometimes also has a short branch to the anal side to the ovary on the lateral zooidal wall (Braem, 1951). A similar branch also might be formed during peristomial budding.

^11^ According to the illustrations of Braem (1951) the proximal funicular cord fuses with a horizontal one/ones(?) in the basal part of *Victorella continentalis*.

^12^ In *Tanganella muelleri* the funicular cord runs from the caecum and almost immediately splits into two branches. One traverses distally towards the ovary on the zooidal wall and the second goes to the pore of the distal septum in the basal part of the zooid. In addition, there may be basal funicular strands going to all four interzooidal septa.

^13^ Similar to *Victorella*, there are proximal (starting from caecum) and basal funicular cords in *Bulbella abscondita*. During zooidal budding from basal zooidal parts attached to the substrate, their basal funicular cord is connected to the zooidal funiculus.

^14^ According to Braem (1940*b*) there are two funicular cords in *Pottsiella erecta*: one starting from the caecum tip which runs down to the zooidal base, and the second starting from the lateral side of the caecum close to the pylorus which runs to the lateral cystid wall. According to Smith *et al*. (2003) in *Pottsiella erecta* the funiculus from the caecum tip has a short branch towards the ovary on the lateral zooidal wall. More proximally, it splits into 2–4 branches (depending on the number of adjacent zooids) in the basal zooidal part, each of which runs to an interzooidal septum. This needs reinvestigation.

^15^ In *Amathia gracilis* the funicular cord from the caecum tip sometimes splits into two branches. One goes distally towards the ovary on the zooidal wall, whereas the second goes to the pore between autozooid and stolon (Braem, 1951). According to Reed (1988) each zooid has two funiculi, the main one runs from the caecum to the septal pore, the second ascends from the septal pore to the ovary on the zooidal wall.

^16^ Muscular elements have been found in the funiculus of *Bugula neritina* (T.F. Schwaha & A.N. Ostrovsky, unpublished data) and possibly are widespread in cheilostomes.
